# Chromosome-level genome assembly of *Tamarindus indica* provides new insights into the evolution of triterpenes and tartaric acid biosynthetic pathway

**DOI:** 10.1186/s43897-025-00222-7

**Published:** 2026-05-13

**Authors:** Mitali Singh, Manohar S. Bisht, Abhijith M.G., Shruti Mahajan, Vineet K. Sharma

**Affiliations:** MetaBioSys Group, Department of Biological Sciences, Indian Institute of Science Education and Research Bhopal, Bhopal, India

**Keywords:** Genome assembly, Tamarind, Whole genome duplication, Tartaric acid, Oxidosqualene cyclase, Triterpenoids

## Abstract

*Tamarindus indica* is the sole member of the genus *Tamarindus* of the Leguminosae family. It is a multipurpose horticultural plant, with every part of the plant finding importance in food, medicine, and other industries. To gain an understanding of genome structure and evolution, we reported the first high-quality genome assembly of *T. indica* anchored to 12 chromosomes with an N50 of 56.6 Mb. Supported by comprehensive transcriptome data, we reported 48,867 protein-coding genes. Through phylogenetic and evolutionary analysis, we uncovered an independent whole-genome duplication event in *T. indica* and highlighted the expression divergence of segmentally duplicated genes and their role in the better adaptivity of the plant. Further, we observed a high expansion of the terpene cyclase mutase (oxidosqualene cyclase, OSCs) gene family and identified nine OSCs and their putative functions in *T. indica*. In addition, by employing integrated genomic, transcriptomic, and metabolomic analysis, we identified the putative L-Idonate dehydrogenase (*L-IDH)* gene in *T. indica* and provided evidence about its possible role in the accumulation of high tartaric acid content in this plant. Our study thus provides an important resource for future genetic and biotechnological studies to understand essential pathways and assist breeding programs for trait enhancements.

**Supplementary Information:**

The online version contains supplementary material available at 10.1186/s43897-025-00222-7.

## Core

We successfully assembled the chromosome-level genome of *T. indica*, providing first insights into its genomic organization, evolutionary history, and WGD event. We further identified key genes involved in the biosynthesis of important phytochemicals, triterpenes, and tartaric acid, that majorly contribute to the medicinal value of the plant. This study thus highlights the significance of *T. indica* as an important horticultural crop and a valuable source of important phytochemicals, emphasizing the need for further genetic research to fully unlock its potential.

## Gene & Accession numbers

Data associated with this study have been deposited at the NCBI SRA database under the BioProject accession PRJNA1217361, BioSample accession SAMN46485370.

## Introduction

*Tamarindus indica,* commonly known as tamarind*,* is a long-lived eudicot plant belonging to the family Leguminosae and a sole member of the genus *Tamarindus *(Saideswara Rao and Mary 2012). Its name originated from the Arabic word “Tamar ul’ Hind,” meaning dates of India. It is believed to be native to Africa and distributed to other tropical Asian countries, especially India, thereby getting its name. It is widely distributed and cultivated across South Asian, African, and American countries (Azad 2018). There are three major varieties of Tamarind: sweet tamarind, sour tamarind, and red tamarind, each offering a unique flavour and biochemical profile of the fruit (Khadivi 2024). The sour variety is the most prevalent, characterized by a high acid content, and is widely used in food for flavour. The sweet variety has low acid and high sugar content, while the red variety is rich in anthocyanins and is used in pharmaceuticals (Khadivi 2024). Breeding programs exist to target the morphological traits like the tree height, stem girth, canopy spread, pod yield, fruits, and floral characteristics, focusing on improving fruit quality, pulp yield, and disease resistance of the plant (Kanupriya 2024; Singh 2025). Almost all parts of the tamarind plant hold significance due to their medicinal, industrial, and culinary uses (Saideswara Rao and Mary 2012; Azad 2018). The plant is renowned for its high nutraceutical value, attributed to the prominent level of dietary fibers, vitamins (like folates, niacin, thiamine, Vitamin A, C, and E), electrolytes (sodium, potassium), and minerals (Azad 2018; Bhadoriya et al. 2011). Additionally, it contains various organic acids (tartaric acid, malic acid, citric acid, etc.) and other phytonutrients (Soni 2019). The phytochemicals found in the different tissues impart several health and medicinal benefits, including antidiabetic, antimicrobial, antioxidant, hepatoprotective, and anti-inflammatory properties (Soni 2019).

Metabolic explorations of the extracts of this plant have shown significant contents of triterpenoids and phytosterols (Bhadoriya et al. 2011). Two important triterpenoids, Lupeol and Lupanone, were detected and isolated from the leaves of *T. indica *(Imam S., et al. 2025). Lupeol content was also found to be high in the flowers and bark of the plant, which imparts anti-microbial and antioxidant properties, contributing to the plant's defense mechanism and overall health (Aly 2022). Besides this, it has emerged as a potent dietary supplement with immense therapeutic value (Sen et al. 2024; Sharma and Gupta 2022). The biosynthetic pathway of these triterpenoids involves a key cyclization step of the precursor 2,3-oxidosqualene, catalyzed by the enzyme oxidosqualene cyclases (OSCs) (Thimmappa 2014). This cyclization step is the critical branch point as it determines whether the pathway leads to the synthesis of sterol or triterpene, depending upon the type of OSC involved. Therefore, studying the OSC gene family is essential for understanding the evolution and diversification of triterpenoid products.

A distinctive property of tamarind is the high accumulation of tartaric acid (TA), up to 12–24% higher than other acidic fruits like grapes and raspberries (Nabavi and Silva 2022; Parvez 2003). TA is one of the most abundant phytochemicals present in tamarind, yet its biosynthetic pathway and specific role in the plant have not been explored much (Walker and Famiani 2018). However, the nutraceutical and pharmacological significance of TA has been reported. It is a naturally occurring organic acid that can work as an antioxidant and laxative and has antimicrobial properties, supporting gut health and aiding food preservation (Nabavi and Silva 2022; Walker and Famiani 2018).

Despite having significant nutraceutical and pharmacological value, *T. indica* remains a highly underutilized plant, with its applications mainly confined to delicacies, flavoring agents, and traditional medicines. The absence of genomic resources limits the exploration and translational application of its essential biosynthetic pathways. In this study, we generated the first chromosome-level genome of *T. indica* (2*n* = 24). We carried out an in-depth analysis of its phylogenomic and gene family evolution dynamics and examined the role of segmentally duplicated genes and their expression divergence. Further, we explored the structural and functional diversification of oxidosqualene cyclase genes, the evolutionary origin of the putative *L-IDH* gene, and its possible role in the high accumulation of tartaric acid, utilizing integrated genome, transcriptome, and metabolome data. These findings will provide advances in both biological and evolutionary perspectives while also elucidating the genome structure and organization of *T. indica.*

## Results

### Genome assembly and quality assessment

**Fig. 1:**
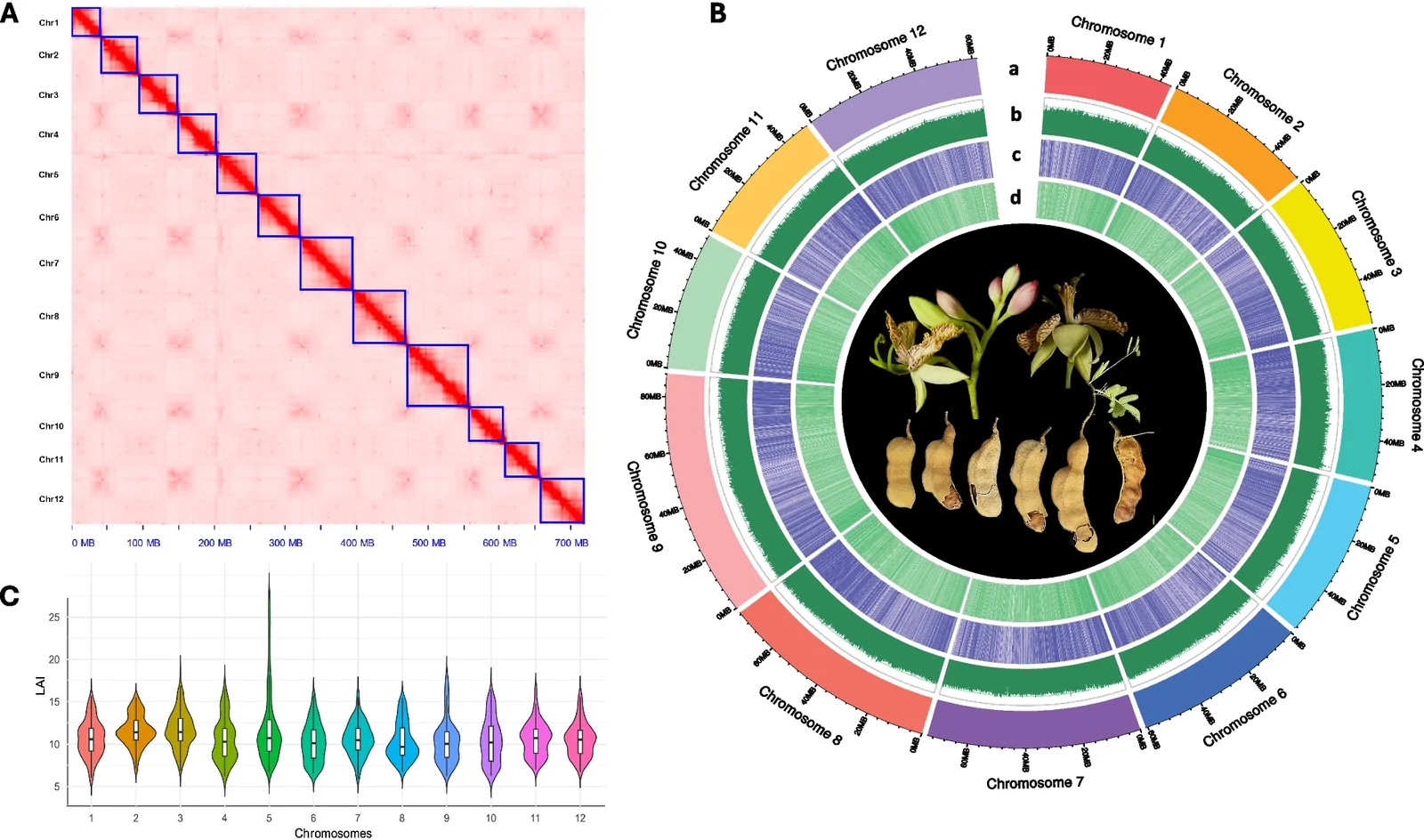
Characteristics of *T. indica* genome assembly. **A** Chromosomal interaction heatmap: Blue frames indicate the 12 assembled chromosomes, **B** Circos plot of *T. indica* genome: The tracks from outside to inside are (a) 12 chromosomes length, (b) GC content, (c) Repeat density, and (d) Gene density. All distributions are drawn in a window size of 100 kb, **C.** LAI scores distribution of 12 chromosomes of *T. indica*

A total of 78 × coverage of linked reads data from 10x Genomics, 28 × of Oxford Nanopore reads, ~ 166 Gb of transcriptome data (~ 10 Gb per tissue), and ~ 200 million paired-end Hi-C reads were generated (Table S1). Through K-mer analysis of the adapter-trimmed 10x Genomics linked reads, the genome size of *T. indica* was estimated to be 778 Mb, with a heterozygosity of 1.12%. The merged assemblies of the 10x Genomics linked reads and Oxford Nanopore long reads resulted in an assembled genome of size 850.4 Mb, with a contig N50 of 4.0 Mb. Further genome scaffolding was carried out using genome-wide chromatin interaction information obtained by sequencing the Hi-C library. The contact map generated from the Hi-C reads resulted in anchoring the genome into 12 superscaffolds (chromosomes) corresponding to ~ 93% of the total assembled genome (Fig. 1A and B). The final genome assembly of *T. indica* was of size 776 Mb, which is near the estimated genome size with a scaffold N50 of 56.6 Mb and the largest scaffold of size 88 Mb (Fig. S1 and Table S2).

For the assessment of the quality and completeness of the genome assembly, the mapping percentage of the raw reads to the final assembly and the BUSCO score of the assembly were calculated. 95.58% of the 10x linked reads, and 95.16% of Nanopore long reads could be aligned to the final genome assembly. The BUSCO completeness score for the final genome assembly was 92.8% (Table S3). Also, the long terminal repeat (LTR) assembly index (LAI) score of all the 12 chromosomes had a value close to or above 10 (Fig. 1C), with the final genome assembly LAI of 10.84, suggesting its quality to be of reference standard.

### Gene set construction and annotation

The de novo repeat library identified 63.85% of repetitive sequences in the genome. Among the interspersed repeats, Gypsy/DIRS1 constituted 25.22%, and Ty1/Copia constituted 10.51% (Table S4). Combining homology and* ab initio* based gene prediction models, a total of 48,867 protein-coding genes were predicted, of which 93.7% could be functionally annotated using publicly available databases (Table S5). The coding genes distribution in COG categories is mentioned in Table S6. Noncoding RNA prediction identified 418 miRNAs, 575 tRNAs, and 796 rRNAs. The mapping of the transcripts to the final genome assembly resulted in alignment ranges from 94 to 85% for different tissues of *T. indica*.

### Phylogenetic tree construction and gene family evolution

**Fig. 2:**
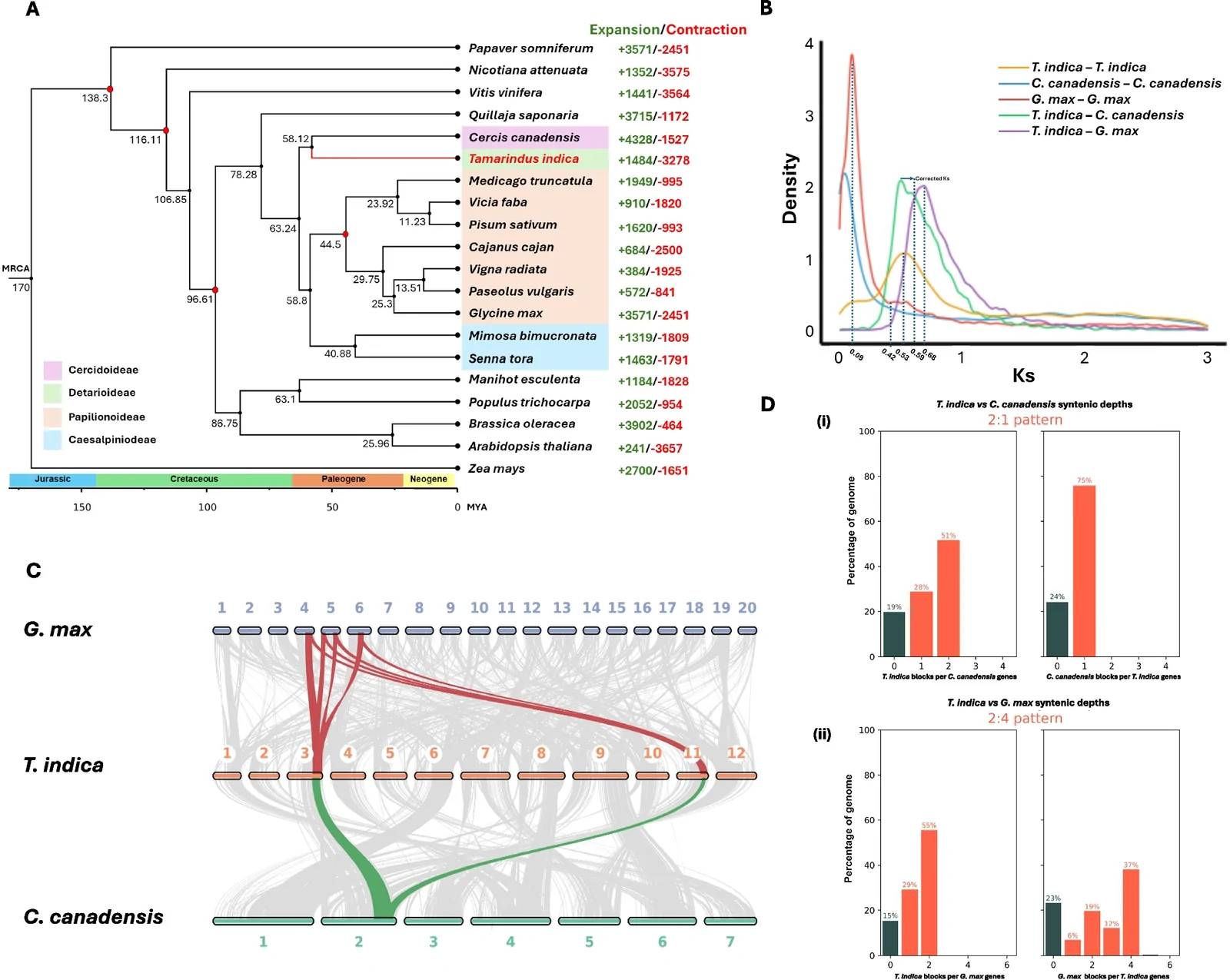
Evolutionary history of *T. indica*. **A** Phylogenetic tree with gene family evolution. Calibrated nodes are highlighted with red dots. MRCA—most recent common ancestor, **B** Synonymous substitution rate (Ks) distributions of paralogs and orthologs of *T. indica*, *C. canadensis* and *G. max,* **C** Macrosynteny between *T. indica*, *C. canadensis* and *G. max*. Two gene blocks in *T. indica* exhibit a single copy in *C. canadensis* (green lines), and one gene block in *T. indica* exhibits four copies in *G. max* (red lines), suggesting that *T. indica* has undergone one independent polyploidization event after the divergence, **D** Syntenic depth (i) *T. indica* vs *C. canadensis* (ii) *T. indica* vs *G. max*

To resolve the phylogenetic position of *T. indica* with respect to legume members, we selected 10 species belonging to the different subfamilies within the Leguminosae (Cercidoideae, Detarioideae, Papilionoideae, and Caesalpiniodeae). The other eight species from the major eudicot lineages and *Zea mays* as an outgroup from monocots were selected to get the divergence time estimation. A total of 531 fuzzy one-to-one orthgroups were identified from the selected 20 plant species and were used for phylogenetic analysis. The phylogenetic tree placed *T. indica* closest to *Cercis canadensis,*consistent with the Legume Phylogeny Working Group (LPWG) (Azani et al. 2017). *Tamarindus* belongs to the Detarioideae subfamily, which diverged early, along with the Cercidoideae subfamily, from the other legumes. Later, it diverged from the Cercidoideae subfamily around the estimated divergence time of 58.12 mya (Fig. 2A). CAFE analysis for the gene family expansion and contraction resulted in the identification of 14,280 filtered gene families across the selected species. Of the 8,328 gene families of *T. indica*, 1,484 gene families were expanded, and 3,278 gene families were contracted. Among the expanded gene families, 12 gene families were highly expanded (> 10 genes) (Supplementary Table 1).

### Whole genome duplication (WGD) and genome synteny of *T. indica*

The Ks distribution of paralogs of *T. indica* showed a recent peak at Ks ~ 0.53, which is after the divergence peak from *G. max* (Ks ~ 0.68) and *C. canadensis* (Ks ~ 0.59), indicating the independent whole genome duplication event in *T. indica* (Fig. 2B). Further, the syntenic depth and whole genome dot plot analysis revealed a 2:4 syntenic ratio with *G. max* and a 2:1 ratio with *C. canadensis*, respectively (Fig. 2C, D, and Fig. S2). These results further support that *T. indica* experienced one independent WGD, *G. max* underwent two independent WGD (Ks ~ 0.42 and Ks ~ 0.09), and *C. canadensis *did not experience any WGD post-divergence (Lee 2024; Schmutz 2010).

Furthermore, intraspecific genome synteny of *T. indica* revealed 27% collinearity. Whereas interspecific genome synteny between *T. indica* and *G. max* was 32% and between *T. indica* and *C. canadensis* was 30% (Fig. 2C).

### Landscape of WGD/Segmental duplicates and their expression divergence in the *T. indica* genome

**Fig. 3:**
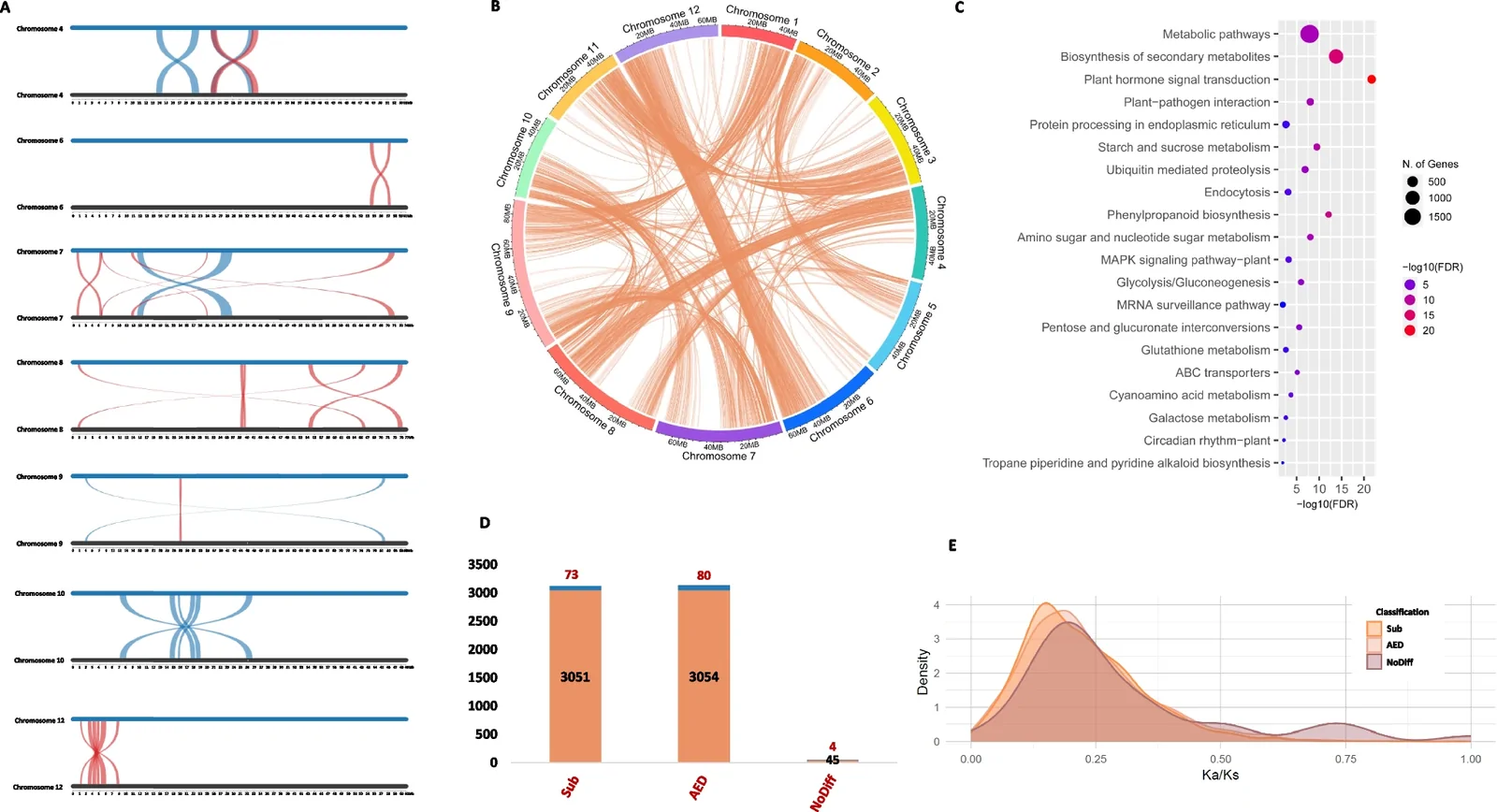
WGD/Segmental duplication and expression analysis. **A** Distribution of intrachromosomal segmental duplication. Blue and red represent forward and reverse matches, respectively, **B** Distribution of interchromosomal segmental duplication, **C** KEGG enrichment of segmental duplicates (adjusted *P-*value < 0.05), FDR: False discovery rate **D** Stacked columns chart showing the ratio of intra to interchromosomal segmental duplicates distribution in AED, Sub, and NoDifff segmental duplicated gene pairs categories, **E** Distribution of Ka/Ks ratio of AED, Sub, and NoDifff segmental duplicated gene pairs. Note: Gene pairs of the chromosome regions are considered

*T. indica*, after divergence, underwent an independent WGD, which resulted in whole genome duplicates/segmental duplicates (here onwards, referred to as ‘segmental duplicates’). These segmental duplicated genes are known to play a crucial role in shaping the genome evolution (Bisht et al. 2024; Sun et al. 2023). In the *T. indica* genome, these genes account for 5.15% of the genome and 21.10% of total protein-coding genes. Further, 97.5% of these segmental duplicates were present between chromosomes (Inter), which was much greater than the percentage of segmental duplicates (3.5%) occurring within the chromosomes (Intra) (Fig. 3A and B). KEGG enrichment analysis revealed that these segmentally duplicated genes were mainly involved in various metabolic pathways, including secondary metabolites biosynthesis, plant hormone signal transduction, plant-pathogen interaction, phenylpropanoid biosynthesis, etc. (Fig. 3C).

WGD in land plants is widely accredited as a key driver of evolution and diversification. The gene duplications arising from WGD provide opportunities for sub-functionalization and neo-functionalization, enhancing adaptability and evolutionary potential (Panchy 2016). Based on the classification criteria (see methodology), of the 6,307 segmentally duplicated gene pairs, 3,124 were categorized as Sub, 3,134 as AED, and 49 as NoDiff class. Further, Sub, AED, and NoDiff classes have 73, 80, and 4 gene pairs found on the same chromosomes (Intra), respectively (Fig. 3D). The Ka/Ks peak value of most of the segmentally duplicated gene pair classes was found to be less than 0.5, which suggests that a significant fraction of these gene pairs arose because of the recent WGD event of *T. indica* and are under strong purifying selection (Fig. 3E) (Supplementary Table 2).

### Genomic diversity and comparative analysis of OSCs in *T. indica*

Triterpenoids are a structurally diverse and biologically significant class of secondary metabolites found in plants. The triterpenoid biosynthesis pathway comprises the terpene backbone synthesis through MVA (mevalonate) and MEP (methylerythritol phosphate) pathways, followed by triterpenoid structural diversification (Chakraborty 2023). During the second stage of triterpenoid biosynthesis, structural diversification occurs when the precursor 2,3-oxidosqualene undergoes cyclization by the 2,3-oxidosqualene cyclase (OSCs) enzymes (Thimmappa 2014; Biswas 2019). The type of OSC involved in cyclization determines whether the pathway leads to sterol or triterpene synthesis, thus representing a critical branch point (Fig. S3). We found the OSC gene superfamily to be highly expanded in the genome of *T. indica*. Through HMM search of the N and C-terminal squalene cyclase hopene conserved domain, 17 candidate OSCs were identified. Nine of these 17 sequences contained the highly conserved DCTA/SE motif and were selected for further analysis. The identified OSCs were designated as TiOSC1 to TiOSC9, with amino acid lengths ranging from 369 to 1,028 residues (Table S7).

**Fig. 4:**
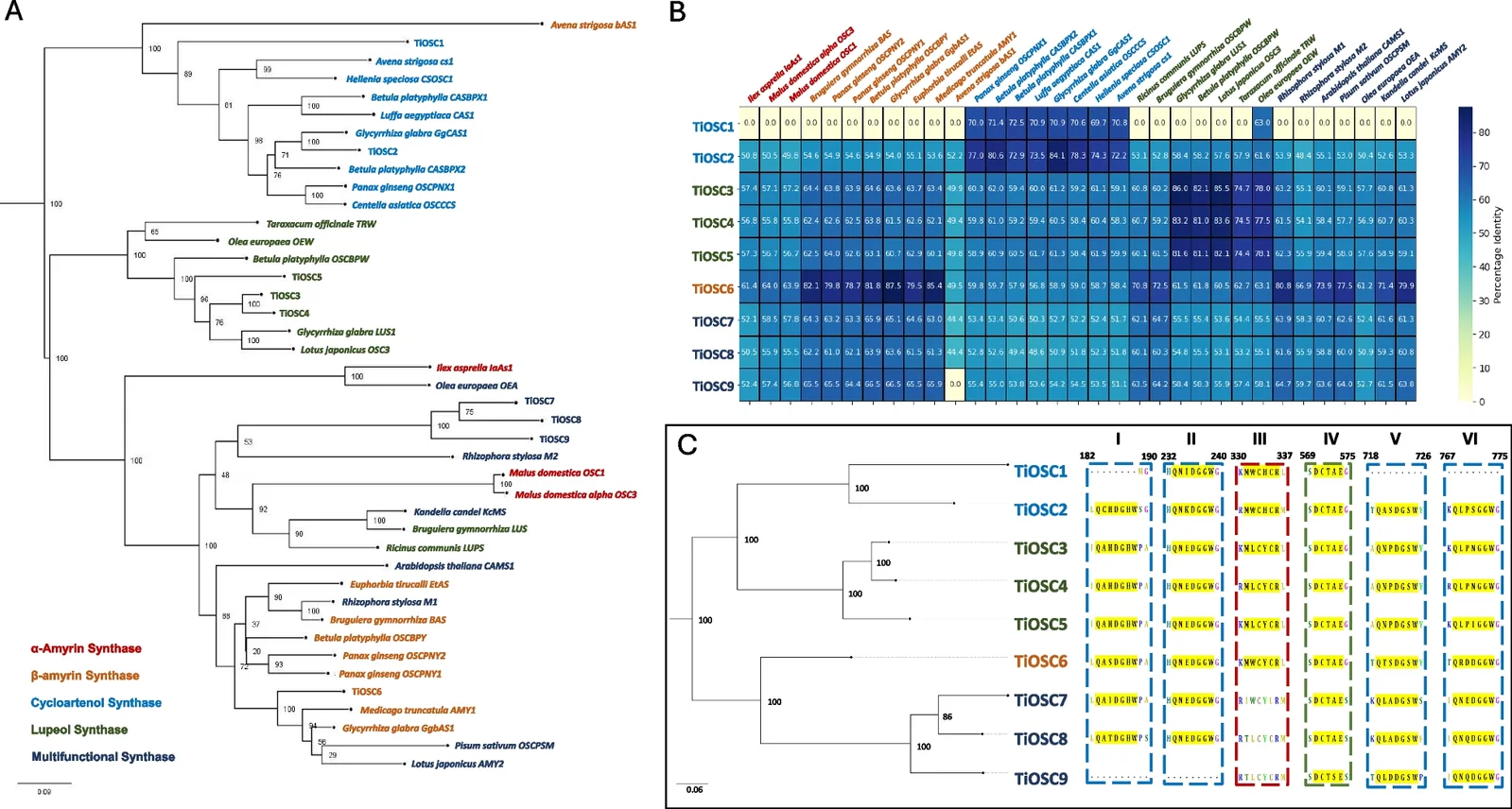
Phylogeny and Sequence analysis of OSCs in *T. indica*. **A** Phylogenetic tree of OSCs of *T. indica* and the functionally characterized OSCs of other plants. The tree was generated using the maximum likelihood method, and the bootstrap support is mentioned on the nodes. Functional categories of the OSCs are distinguished with color codes, **B** Amino acid sequence identity heat map of TiOSCs amino acid against OSCs of other species mentioned on the top, **C** Key motifs identified in the amino acid sequences of the OSCs of *T. indica*, Box IV shows the DCTA/SE motif; BOX I, II, V and VI are the QW repeat motifs; Box III indicates the MW/LCY/HCR motif

The phylogenetic analysis of the OSCs of *T. indica* with the functionally characterized OSCs from other species placed them into separate clades representing their putative functional class (Fig. 4A). TiOSC1 and TiOSC2 formed a clade with the cycloartenol synthase-type OSCs of other plants. TiOSC3, TiOSC4, and TiOSC4 formed a clade with the lupeol synthase type OSCs responsible for synthesizing lupeol-type triterpenoids in plants. TiOSC6 was positioned close to the β-amyrin synthase type OSCs, and the other three OSCs were grouped and positioned near the α-amyrin type and multifunctional OSCs. Further, these OSCs of *T. indica* showed more than 70% amino acid identity with their assigned functional categories as per phylogeny (Fig. 4B).

In addition to the highly conserved DCTAE motif, seven of the nine OSCs (TiOSC2–TiOSC8) contained four QXXXGXW motifs essential for stabilizing the carbocation during the cyclization process, whereas TiOSC1 and TiOSC9 possessed two of these QW motifs (Fig. 4C) (Du 2024). Furthermore, the genes categorized into functional categories based on the phylogeny were validated by screening the class-specific conserved motifs. The β-amyrin synthase-specific conserved motif MWCYCR was identified in TiOSC6. While TiOSC3, TiOSC4, and TiOSC5, assigned to the lupeol synthase OSC category, had the MLCYCR motif at the same position. The substitution of W with L in this motif facilitates the stabilization of the lupenyl cation, as confirmed by mutational studies (Du 2024; Chen 2021), enabling lupeol synthesis. Genes TiOSC1 and TiOSC2 exhibited the MWCHCR motif, which has been implicated in supporting the formation of the protosteryl cation intermediate during cycloartenol synthesis (Poralla et al. 1994).

### Identification and expression analysis of SDH homologs in *T. indica*

The TA biosynthesis pathway involves the conversion of ascorbic acid to idonic acid, which is then transformed into 5-keto-D-gluconic acid and, with subsequent cleavage, yields TA. Kinetic analysis of the intermediates suggests the L-idonate to 5-keto D-gluconate transformation as a rate-limiting step of the pathway, catalyzed by the enzyme L-idonate dehydrogenase (*L-IDH*). It is the only enzyme characterized and confirmed to be involved in TA synthesis, which shares a close homology with plant sorbitol dehydrogenase (*SDH*). A comprehensive work on the *SDH* homologs across different plant species, conducted by Jia et al., reveals *L-IDH* to be a Class II *SDH* (Jia 2015). However,*T. indica*, which contains a significantly high amount of TA (8–18%) (Shankaracharya 1998; Santhosh 2013), was not included in this study due to the unavailability of its genomic data, prompting further investigation into its *SDH* homologs.

**Fig. 5:**
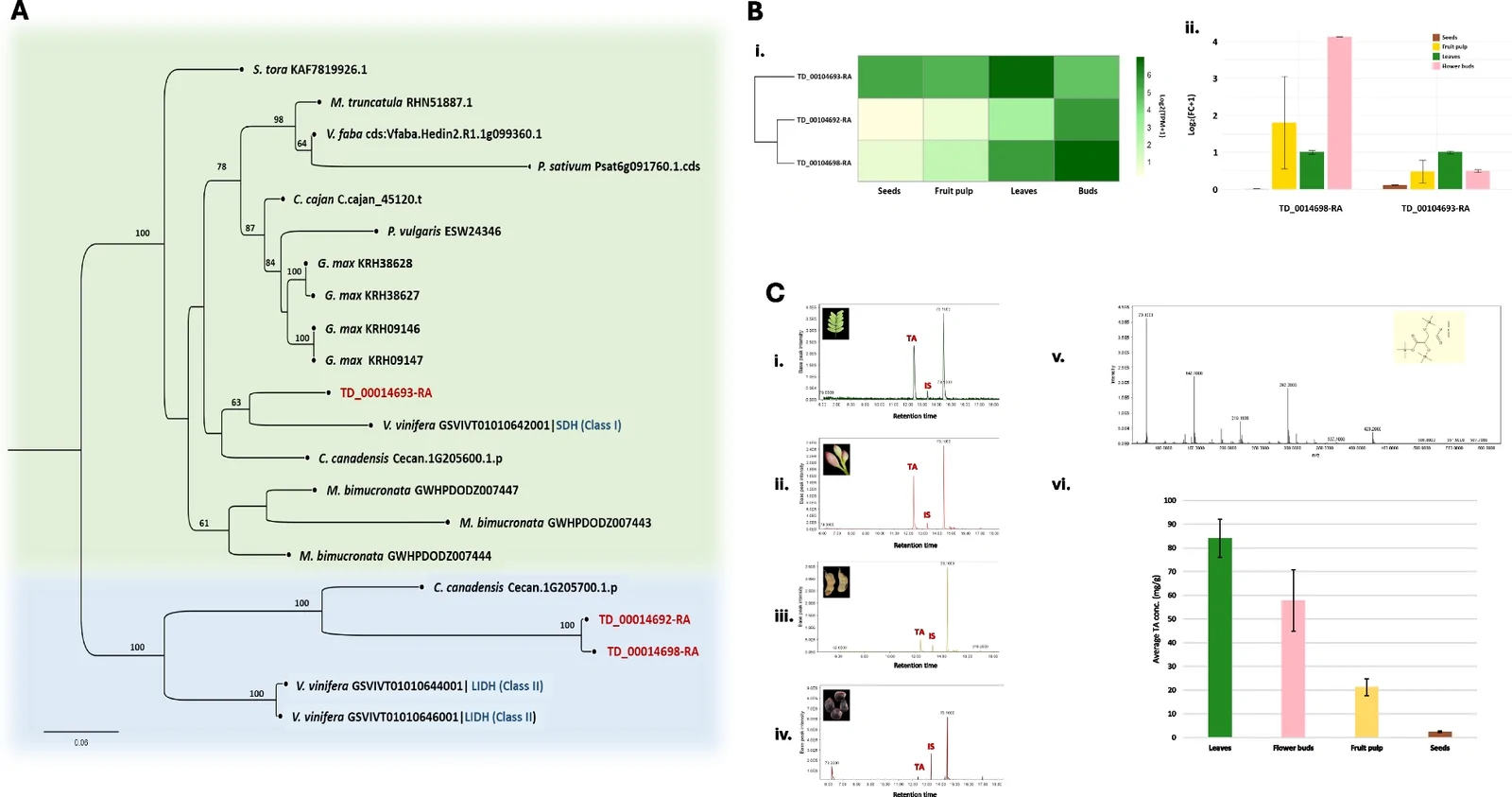
Phylogenetic and gene expression analysis of *SDH* Genes and metabolite profiling of tartaric acid in *T. indica*. **A** The maximum likelihood phylogenetic tree of the *SDH* homolog genes identified in the Legumes species and *Vitis vinifera* (with the *SDH* class marked in blue). *SDH* homologs of *T. indica* are marked in red. Bootstrap values (above 50) are displayed on the nodes, **B** (i) Heat map of the expression value in Log_2_(TPM + 1) of the three *SDH* homologs of *T. indica* in the four tissues (*n* = 2) (ii) RT-qPCR analyses of identified *SDH* homologs (TD_00140698-RA and TD_00104693-RA) across four tissues (*n* = 3 biological replicates). Expression levels were normalised using *Elongation factor1-alpha (EF1-α)* as the endogenous control. Gene expression is shown as fold change relative to leaves (reference tissue). Error bars represent the standard error mean (± SEM), **C** GC–MS analysis of the extracts of different tissues of tamarind: (i-iv) Total Ion Chromatogram (TIC) showing tartaric acid-TMS derivative (TA) peak at retention time 12.3–12.5 min and internal standard (IS) peak at 13.3–13.4 min. Peak intensity is mentioned on the y-axis. v. Mass spectra of TA-TMS derivative. vi. The bar plot representation of the TA concentration in each tissue in mg/gram dry weight of the tissue

Three *SDH* genes were identified in the genome of *T. indica*. Two of these sequences (TD_00014692-RA and TD_00014698-RA) were highly identical, with an amino acid sequence identity of 98.17%, and the third gene sequence, TD_00014693-RA, shared 71.26% and 70.85% with TD_00014692-RA and TD_00014698-RA, respectively (Fig. S4). The phylogenetic analysis of these three sequences with the *SDH* sequences identified from the other legume species and the three SDHs of *Vitis vinifera* classified them into two separate clades (Fig. 5A). TD_00014692-RA and TD_00014698-RA of *T. indica* formed a clade with the Class II *SDH* or *L-IDH* gene of *V. vinifera* (GSVIVT01010646001 and GSVIVT01010644001). Whereas TD_00014693-RA was positioned closer to the Class I *SDH* of *V. vinifera,* along with the identified *SDH* homologs in the other legume species.

Further, the expression pattern of Class I *SDH* genes of *T. indica* was found to be different from the Class II *SDH* gene. The identified Class I *SDH* (TD_00014693-RA) had a uniform expression in all four tissues. In contrast, the identified Class II *SDH* (TD_00014692-RA and TD_00014698-RA) showed different expression levels in these tissues, with the highest TPM value observed in flower buds and leaves and the least in seeds (Fig. 5B i).

The RT-qPCR analysis further validated the differential expression patterns of Class I and Class II *SDH* genes as observed in the RNA-seq data. TD_00104698-RA (Class II *SDH*) exhibited significantly higher expression levels in flower buds, while very low expression in the seeds. In contrast, TD_00104693-RA (Class I *SDH*) showed a relatively uniform expression across the four tissues (Fig. 5B ii), with evident expression in seeds as well.

The GC–MS analysis of the metabolites extracted from the tissues revealed several primary metabolites like carbohydrates, sugar alcohols, organic acids and fatty acids (Supplementary Table 3–6). A prominent TA peak was detected at a retention time of 12.3–12.5 min in leaves, flower bud, and fruit pulp extract, while this peak intensity was very low in seed extract (Fig. 5C i-v). The average concentration of TA calculated in mg/gram dry tissue weight was highest in leaves (Avg. 84 mg/g dry weight) and flower buds (57 mg/g dry weight) compared to fruit pulp (21 mg/g dry weight) and seeds (2 mg/g dry weight) (Fig. 5C vi).

## Discussion

*Tamarindus indica* is a significant horticultural crop with a wealth of pharmacologically important metabolites and nutraceuticals. However, the lack of a reference genome limits the genetic studies, the investigation of important genes and key biosynthetic pathways of this plant. Thus, in this study, we report a high-quality chromosome-scale genome assembly of *T. indica*, with an N50 of 56.6 Mb and an LAI score of 10.84, indicating the assembly to be of reference standard. The genome was found to have 1.12% heterozygosity with 63.85% repetitive sequences.

*T. indica* belongs to the third largest angiosperm family, the Leguminosae. Previously, it was placed in the Caesalpinoideae subfamily, but in 2017, the Legume Phylogeny Working Group revised the higher-level classification of the legume family and formed Cercidoideae, Detarioideae, Dialioideae, and Duparquetiodeae as distinct subfamily and placed *T. indica *into Detarioideae (Azani et al. 2017). Our phylogenetic analysis also placed *T. indica* in a separate clade from the Caesalpinoideae subfamily and closest to *Cercis canadensis* of the Cercidoideae subfamily.

Ks analysis revealed that *T. indica* experienced an independent WGD after splitting from Cercidoideae (*C. canadensis*) at Ks peak ~ 0.53. Synteny analysis corroborates these findings, with a 2:1 syntenic pattern observed between *T. indica* and *C. canadensis*, indicative of a recent WGD event in *T. indica* and the absence of any independent WGD in *C. canadensis* post divergence, which is also reported in the recent genomic studies of the genus *Cercis *(Lee 2024; Li 2023). The resulting gene duplicates from WGD confer genes with potential sub-functionalization and neo-functionalization, contributing to better adaptivity of the plant. The *T. indica *genome revealed 6,307 gene pairs with segmental duplications, with over half categorized as sub-/neo-functionalized pairs, while nearly the other half exhibited asymmetric expression patterns. Both categories exhibit genes involved in key pathways, such as plant hormone signalling, carbon fixation, plant-pathogen interaction, etc., mediating plant growth and resilience with biotic and abiotic stresses (Table S8-9). Multiple duplicated copies of these functionally essential genes involved are potentially associated with ecological adaptations, for example in functional pathways related to plant growth (i.e., plant hormone signal transduction), both TIR1, which is an F-box protein responsible for the reception of auxin, and its target AUX/IAA proteins were found to be segmentally duplicated (Li et al. 2021; Jiang 2018). In light of these findings, our results suggested an evolutionary fitness advantage to *T. indica*, offered by these duplicates arising from the recent WGD event.

Triterpenoids are a large and structurally diverse class of natural compounds widely found in plants. In addition to their specialized roles in plant communication and biotic and abiotic stress tolerance, these triterpenoids have been extensively studied for their considerable therapeutic values (Biswas 2019; Li 2023; Gill 2016). The cyclization of the 30 Carbon 2,3-oxidosqualene is the key step in triterpenoid diversification, catalyzed by the enzymes of the oxidosqualene cyclase (OSCs) family (Thimmappa 2014; Han 2022; Yang et al. 2023). In our study, we found a high expansion of the OSC gene family and identified nine OSCs in the genome of *T. indica*. These OSCs were categorized into five major classes: cycloartenol synthase (precursor for phytosterols synthesis), β-amyrin synthase, lupeol synthase, α-amyrin synthase, and multifunctional synthases (Yang et al. 2023; Brendolise 2011). Three of the nine OSCs were categorized as lupeol synthase, which synthesizes lupeol, a prominent triterpenoid found in *T. indica *(Aly 2022; Al-Fatimi et al. 2007). Lupeol is a versatile compound with significant therapeutic values, including anticancer, anti-inflammatory, antiviral, and antimicrobial properties (Sen et al. 2024; Sharma and Gupta 2022). The identification of these OSCs and their deduced amino acid sequence will be helpful for understanding the metabolic pathways of triterpenoid synthesis and their diversification in *T. indica*. It also lays the foundation for future biotechnological research for trait enhancement and exploration of the industrial applications of important triterpenoids from *T. indica*, facilitating better utilization of this plant beyond its culinary applications.

Tartaric acid (TA) is the major phytochemical and the highest among all the organic acids present in tamarind. The high accumulation of TA (8–18%) is presumably linked to offer several adaptive advantages to the plant. It contributes to the allelopathic potential of *T. indica*, enhancing its competitive ability in the natural ecosystem (Parvez 2003). Studies have also shown that TA delays germination, allowing seeds to get dispersed to distant sites (Bhattacharya 2021). Major studies on TA biosynthesis have mainly been restricted to Vitaceae and Geraniaceae family members, while Leguminosae received minimal attention despite *T. Indica *being the most prominent candidate for TA accumulation (Burbidge 2021; Narnoliya 2018; DeBolt et al. 2006). The studies revealed *L-IDH* as a crucial gene involved in the pathway, later classified as a Class II *SDH *(Jia 2015; Burbidge 2021; Narnoliya 2018). Our study found three *SDH* homologs in the genome of *T. indica*, of which two shared close homologies with the *L-IDH* gene of *Vitis vinifera*. In contrast, the *SDH* homologs from the other legume species were classified as Class I *SDH*. The identification of the Class II (putative *L-IDH)* gene in *T. indica* and its close homology with the evolutionary distant *Vitis’ L-IDH *suggests its potential involvement in TA accumulation similar to that found in Vitaceae members (Burbidge 2021). The RNA-seq analysis revealed differences in the expression pattern of Class I and Class II *SDH* genes across the different tissues analysed, suggesting their functional divergence in *T. indica.* TD_00014693-RA, representing a Class I *SDH*, displayed a nearly uniform expression across the four tissues, indicating a possible role in maintaining basal levels of sorbitol metabolism. In contrast, the Class II *SDH* genes, TD_00014692-RA and TD_00014698-RA, exhibited tissue-specific expression patterns, with notably higher transcript abundance in flower buds and leaves, and significantly lower levels in seeds. This trend was further validated by RT-qPCR, particularly for TD_00104698-RA (Class II *SDH*), which showed an elevated expression in flower buds compared to seeds. Furthermore, the tartaric acid quantification in these tissues also revealed a similar pattern, showing the highest TA concentration in leaves and flowers, while the minimum concentration was detected in the seeds. These observations hint towards the potential role of these Class II genes in TA accumulation in *T. indica.* However, further functional characterization and enzymatic assay are required to elucidate the precise roles of these isoforms.

In conclusion, our work presents the first high-quality genome assembly of *T. indica,* providing insights into its evolutionary history and genome duplication. The identification of recent WGD and the analysis of segmental duplicates highlights the adaptive potential of the plant. Furthermore, the insights into the biosynthetic pathways of the two essential phytochemicals, triterpenoids and tartaric acid, can aid in biotechnological research to target these pathways for enhancing the nutraceutical and pharmaceutical value of the plant. As *Tamarindus* is a monotypic genus, this study holds significance by offering comprehensive genomic clues that contribute to the unique traits of the plant and lay the foundation for future exploration.

## Methodologies

### DNA and RNA extraction and sequencing

Leaves of the plant were collected from the tree growing in the campus of IISER Bhopal (23.2˚ N 77.2˚ E). Species confirmation was done using the molecular barcodes ITS and matK. High molecular DNA was extracted from fresh leaves using CTAB lysis buffer, as described in Bisht et al. (Bisht et al. 2024). DNA purity and concentration were checked using NanoDrop™ 8000 Spectrophotometer (ThermoFisher Scientific, USA) and Qubit 2.0 fluorometer. The 10x genomic library was prepared on the Chromium Controller instrument (10x Genomics, CA) and sequenced on the Illumina Novaseq 6000 platform to generate 60 Gb data. For Nanopore sequencing, a few rounds of purification and size selection were performed using AMPure XP magnetic beads (Beckman Coulter, USA). DNA with optimum quality was taken forward for library preparation using the ligation kit SQK-LSK110 (Oxford Nanopore), and sequencing was performed on flowcell FLO-MIN106 on the MinION Mk1C platform (MinKNOW software version 22.08.6).

For RNA extraction, leaves and fruits were collected in February, a few weeks before the onset of ripening of the fruit. The leaf, fruit pulp, and seeds were immediately transferred to the laboratory, flash frozen, and stored at −80˚C till further processing. The flowers were collected in April, flash-frozen, and stored at −80˚C. For the RNA extraction, 1 g tissue (three biological replicates for each tissue, except two for fruit pulp) was crushed in liquid nitrogen and transferred to 10 ml prewarmed lysis buffer (300 mM Tris HCl, 25 mM EDTA, 2 M NaCl, 2% CTAB, 2%PVP). 2% beta-mercaptoethanol was added to the lysis buffer just before the lysis procedure. Tissues were lysed for 15 min at room temperature with intermittent mixing by inverting the tubes every 3–4 min. Two rounds of chloroform: isoamyl alcohol extraction was performed, followed by the addition of 0.3 × volume of sodium acetate (3 M, pH = 5) and 0.6 × volume ice-cold isopropanol. The tube was kept at −20˚C for efficient precipitation, and the pellet was collected after 3 h by centrifugation at 5000 g for 30 min at 4˚C. The pellet obtained was washed with 70% ethanol and suspended in 1 ml Nuclease-free water. 0.3 × Volume of 8 M LiCl was added, followed by overnight incubation at −20˚C for RNA precipitation. RNA was pelleted by centrifugation at 20,000 g for 30 min at 4˚C and eluted in NFW after ethanol washing. Further purification of the RNA was done using the Qiagen RNeasy mini kit spin column following the manufacturer’s protocol, with on-column DNA digestion (Mahajan 2024). The RNA quality was assessed on NanoDrop 8000 Spectrophotometer (ThermoFisher Scientific, USA), and quantification was done on Qubit 2.0 fluorometer using Qubit RNA BR Assay kit (Invitrogen, USA). Final libraries were quantified and insert size was determined using Tapestation 4150 (Agilent) utilizing sensitive D1000 screencaps (Agilent). The library with passed QC was taken forward for sequencing on NovaSeq 6000, generating ~ 10 Gb data per tissue.

### Hi-C library preparation and sequencing

The Hi-C library was prepared with young and freshly collected leaves of the plant using the EpiTect Hi-C kit (Qiagen), following the manufacturer’s protocol. The crushed leaves were fixed with formaldehyde to crosslink the chromatin inside the nucleus. Following cell lysis, intact nuclei were collected and digested using the Hi-C digestion solution provided in the kit. Sticky ends were labelled and ligated, followed by de-crosslinking and DNA purification. The extracted DNA was quantified and fragmented to 450–500 bp length. The labelled Hi-C fragments were captured via streptavidin beads pulldown. Subsequent library preparation was carried out using the standard procedure of end-repair, phosphorylation, and dA tailing of Hi-C fragments. After Illumina adapter ligation, the insert size of the prepared library was determined on Tapestation 4150 utilizing high-sensitive D1000 screentapes (Agilent). The sequencing was done on the Illumina NovaSeq 6000 platform.

### Data preprocessing and Genome-size estimation

The barcoded 10x Genomics linked reads were trimmed using the proc10xG python scripts (https://github.com/ucdavis-bioinformatics/proc10xG) to remove the barcode sequences. Nanopore reads were basecalled using Guppy v3.2.1 (Oxford Nanopore Technologies), and adapters were trimmed using Porechop v0.2.4 (Oxford Nanopore Technologies).

The 10x trimmed reads were used for genome size estimation of *T. indica *using k-mer-based analysis. The k-mer frequencies (m = 21) of the raw reads were calculated using the Jellyfish v2.3.1 (Marçais and Kingsford 2011) and used for the estimation of genome characteristics using GenomeScope2.0 (Ranallo-Benavidez 2020) (Fig. S1).

### Genome assembly and quality assessment

The 10x linked reads were assembled using Supernova v2.1.122 with the ‘maxreads = all’ parameter. The assembly was corrected with the help of barcode-filtered reads (https://support.10xgenomics.com/genome-exome/software/pipelines/latest/installation) using Tigmint v1.2.6 (Jackman 2018)and scaffolded using ARCS v1.1.1 (Yeo et al. 2018)with default parameters. Adapters-free nanopore reads were used to further scaffold the genome assembly using LINKS v1.8.6 (Warren 2015) with default parameters.

Long read assembly of the pre-processed Nanopore reads was performed using Flye v2.9.1 (Kolmogorov et al. 2019) with default parameters. The barcode-flitered 10x Genomics linked reads were used for polishing (three iterations) and scaffolding the long-read assembly using Pilon v1.23 (Walker 2014)and ARCS v1.1 (Yeo et al. 2018), respectively. Nanopore long reads were used for further scaffolding of the assembly using LINKS v1.8.6 (Warren 2015). The constructed 10x Genomics assembly and Nanopore assembly of*T. indic*a were merged using Quickmerge v0.3 (Chakraborty et al. 2016; Chakraborty 2021)to achieve a more contiguous assembly. LR_Gapcloser (Xu et al. 2018)was used in five iterations for closing the gaps in the merged assembly using adapter-free long reads, and the final polishing of the assembly was performed using Pilon v1.23 (Walker 2014). Purge Haplotig v1.1.270 (Roach 2018) was used to remove redundant heterozygous regions responsible for increased assembly length.

To obtain the chromosome-level genome assembly of *T. indica*, clean Hi-C paired-end reads were aligned to draft assembly using BWA (Li 2009) and a deduplicated list of Hi-C reads was generated using the Juicer pipeline, and the draft assembly was scaffolded using 3D-DNA (Durand et al. 2016). The resulting assembly was then visualized and manually curated using Juicebox v1.11.08 software (Durand 2016). The gaps between the pseudochromosomes were then filled by TGS-Gapcloser v1.2.1 (Xu 2020) by two rounds using long-read data.

The pre-processed raw 10x linked reads and Nanopore reads were mapped to the assembled genome using BWA-MEM (v0.7.17) (Li and Durbin 2009), Minimap2 (v2.17) (Li 2018), respectively, and the mapping statistics were calculated using SAMtools (v1.9) (Li et al. 2009) “flagstat” utility, to validate the quality of the assembly. Further, the completeness of the genome assembly was analyzed through Benchmarking Universal Single-Copy Ortholog (BUSCO) v5.4.3 (Manni et al. 2021) with the embryophyta_odb10 database. Also, the LTR Assembly Index (LAI) was calculated to assess the continuity of Long Terminal Repeat retrotransposons (LTR-RTs) in the *T. indica *Genome using LTR_retriever v2.9.0 (Ou and Jiang 2018).

### Gene set construction and annotation

The repetitive sequences in the genome of *T. indica *were identified, and a de novo repeat library was constructed using RepeatModelar v2.0.3 (Flynn et al. 2020). The redundant sequences were clustered using CD-HIT-ESTv4.8.1 (Li and Godzik 2006) with a seed size of 8 bp and a sequence similarity threshold of 90%. The obtained repeats were soft masked in the genome assembly using RepeatMasker v4.1.2 (http://www.repeatmasker.org), and the masked genome assembly was then used for gene set construction of the tamarind genome using the MAKER pipeline (Cantarel et al. 2008). A combination of evidence-based and *ab initio* gene prediction models was used in the MAKER pipeline in three iterations. Transcriptome assembly of *T. indica *was constructed from the quality filtered RNAseq reads of different tissues (leaf, fruit pulp, seed, flower bud) using Trinity v2.14.0 (Haas et al. 2013). This de novo transcriptome assembly of tamarind and protein sequence of 12 Leguminosae species (*Acacia crassicarpa, Cajanus cajan, Glycine max, Lupinus angustifolius, Medicago truncatula, Mimosa bimucronata, Phaseolus vulgaris, Pisum sativum, Senna tora, Trifolium pratense, Vicia faba,* and *Vigna radiata*) (Table S10) were used as empirical evidence for the gene set construction using BLAST (Altschul et al. 1990) and Exonerate v2.2.0 (https://github.com/nathanweeks/exonerate). In the last two rounds of the MAKER pipeline, AUGUSTUS v3.2.3 (Stanke 2006) and SNAP v1.0 (Korf 2004) were used for *ab initio* gene prediction. The MAKER-derived gene set obtained was further refined based on Annotation Edit Distance (AED) value and length-based criteria, keeping genes with AED value < 0.45 and length > 150 bp to obtain the final high-confidence gene set (Jaiswal 2021).

### Phylogeny and gene family evolutionary analysis

For the phylogenetic tree construction and analysis of the evolutionary pattern of gene families in *T. indica*, protein sequences of 10 legume species and eight species from other representative taxa were used, with *Zea mays *as an outgroup (Table S10). The longest isoform of each protein was selected. OrthoFinder v2.5.4 (Emms 2019) was used to construct the orthogroups, followed by KinFin v1.2 (Laetsch 2017) to extract the fuzzy one-to-one orthogroups, which were individually aligned using MAFFT v7.467 (Katoh 2002). The empty sites from the multiple sequence alignment were removed using BeforPhylo v0.9.0 (https://github.com/qiyunzhu/BeforePhylo), and a maximum likelihood phylogenetic tree was constructed using RAxML v8.2.12 (Stamatakis 2014) with the ‘PROTGAMMAAUTO’ amino acid substitution model and a bootstrap value of 100. Species divergence was estimated with MCMCtree implemented in the PAML package v4.10.6 (Yang 2007) using four calibration points obtained from the TimeTree database v5.0 (https://timetree.org/) (Table S11).

The gene family expansion/contraction analysis was performed by CAFE v5 (Mendes et al. 2020; Mahajan 2023). An All-versus-All mode BLASTP was performed on the longest isoforms protein sequences, and the results were clustered using MCL v14-137 (Enright et al. 2002). Filtering of gene families with genes from < 2 species and gene families containing ≥ 100 gene copies for ≥ 1 species was done (Chakraborty 2022). The constructed species tree was converted to an ultrametric tree using r8s software (Sanderson 2003) with a divergence estimate of 117 MYA (Million Years Ago) between *T. indica* and *V. vinifera* obtained from the TimeTree database (https://timetree.org/). Two-lambda (λ) model (birth–death rate) of CAFE (Mendes et al. 2020) was used to analyze the evolution of gene families using the filtered gene families output and the ultrametric species tree. Gene families with ≥ 10 genes among the expanded gene families were considered highly expanded.

### Analysis of whole genome duplication (WGD) and genome synteny

The Ks (Substitutions per synonymous site) distribution analysis was performed to estimate WGD events in *T. indica, C. canadensis,* and *G. max *using wgd v2.23 (Chen 2024). Further, estimates of differences in synonymous substitution rates among the lineages of the species were performed using the wgd “ksd” utility, and an adjusted Ks plot was generated. The intragenomic and intergenomic synteny was performed by MCScanX (Wang et al. 2012) with at least five gene pairs required per syntenic block, and JCVI (Tang 2024) was employed to perform syntenic depth calculation, whole genome dot-plot, and macrosynteny visualization.

### Identification of WGD/Segmental duplication and expression analysis

The WGD/segmental duplicate genes were identified using the duplicate_gene_classifier implemented in MCScanx (Wang et al. 2012). Further, collinear gene pairs present in the chromosome regions were obtained from the MCScanx result performed during synteny analysis. The TPM (transcripts per million) values of the segmentally duplicated gene pairs were calculated using Kallisto v0.48.0 (Bray et al. 2016) for each tissue. The gene pairs based on their expression (TPM value) were then classified into three categories: (i) sub-/neo-functionalized pairs (Sub), where each duplicate showed higher expression than the other in at least one sample; (ii) asymmetrically expressed duplicates (AED), where one duplicate exhibited higher expression in at least one-third of the samples and was never expressed lower than its partner in any sample; and (iii) the remaining pairs, categorized as no-difference (NoDiff) pairs (Sun et al. 2023; Lan 2016). The numbers of non-synonymous substitutions per synonymous site (Ka), synonymous substitutions per synonymous site (Ks), and the Ka/Ks ratios were calculated using KaKs Calculator 3.0 (Zhang 2022).

### Identification, phylogenetic, and sequence analysis of OSC genes

The identification of putative genes of oxidosqualene cyclase (OSC) was performed using the homology search of two conserved domains, the N- squalene-hopene cyclase (PF13249) and C- squalene hopene cyclase (PF13243), using HMMER (Finn 2011) with an E-value 1e^−5^. The identified OSCs in the genome of *T. indica *were filtered based on the presence of a conserved ‘DCTA/SE’ motif (Haralampidis 2001; Yang 2024). The protein sequences of functionally characterized OSCs from other plant species were downloaded (Table S12). These OSCs belong to five major functional categories based on the product they synthesize: cycloartenol synthase, β-amyrin, α-amyrin, lupeol synthase, and multifunctional OSCs (synthesize a combination of triterpenoid products). The obtained sequences were aligned along with the putative OSCs of *T. indica *using CLUSTALW (Multiple 2025), and a Maximum Likelihood tree was constructed using RAxML (Stamatakis 2014) with the PROTGAMMAJTT substitution model. The branch support of the phylogenetic tree was computed using 100 bootstraps. For the motif search, the putative OSCs of *T. indica *were aligned using CLUSTALW (Multiple 2025), and the motifs were visualized using MEGA 11 software (Tamura et al. 2021).

### In silico identification and characterization of SDH homologs

Protein sequences of the three sorbitol dehydrogenase genes found in *Vitis vinifera*, classified as Class I *SDH* (GSVIVT01010642001) and Class II *SDH*(GSVIVT01010646001 and GSVIVT01010644001) were downloaded (Jia 2015). The three *SDH* genes were used as queries against the protein sequences of the Leguminosae species used in phylogenetic analysis, including the protein sequence of *T. indica*. BLASTP hits with an identity of 60% and an E value cutoff of 10^–5^ were selected for each species. The identified putative *SDH* homolog sequences from all species and *V. vinifera *were aligned using MAFFT (Katoh 2002), and a maximum likelihood tree was constructed using RAxML (Stamatakis 2014).

### RT-qPCR of SDH homologs

RNA extracted from the four tissues (buds, fruit pulp, seed, and leaves) was purified using the RNeasy mini kit (Qiagen), with on-column Ambion DNase I (Invitrogen) treatment. Three biological replicates were used for each tissue. 1 µg of RNA was used for cDNA construction using the iScript cDNA Synthesis Kit (BioRad). One tenth of the constructed cDNA was used for the RT-qPCR using the iTaq Universal SYBR Green Supermix (BioRad) on a QuantStudio 5 system (Applied Biosystem). *Elongation factor-1 alpha* (*EF1-α*) was used as an endogenous control, and fold change was calculated using the 2^^(−ΔΔCt) ^method (Livak 2001)with leaf as reference sample. All primer sequences are listed in Table S14.

### Extraction of metabolites and GCMS analysis

Untargeted metabolite analysis was performed using GC–MS across the four tissues following the method described by Lisec et al. (Lisec 2006). 100 mg of the freeze-dried tissue was used with an added sonication step for 30 min at room temperature for the extraction of metabolites. Polar extracts were aliquoted into tubes, vacuum dried, and derivatized for running on GC–MS. The GC–MS conditions and the parameters for peak analysis are mentioned in Table S13. The raw files (.cdf) were pre-processed with the MZmine software v3.9.0, followed by library search using the GNPS server (Wang et al. 2016; GNPS 2025).

For tartaric acid quantification in different tissues of *T. indica*, metabolites were extracted from 10 mg of lyophilized tissues. During extraction, 12 µl of ribitol (2 mg/ml, TCI) was added as internal standard (IS) to each sample. 100 µl of the polar extracts (upper phase) obtained were aliquoted into fresh tubes and vacuum-dried. The dried metabolites were derivatized using methoxyamine hydrochloride (Sigma) solution in pyridine (Sigma) (20 mg/ml) and then with N,O-bis(trimethylsilyl)trifluoroacetamide reagent (TCI). The derivatized samples were analyzed on an in-house Agilent 7890 A GC with a 5975 C MS system. Raw files (.cdf) were obtained and analyzed using the MZmine software v3.9.0 (Schmid 2023). The peaks of TA and IS were identified, and their relative peak area was calculated. A calibration curve was generated using calcium meso-tartrate trihydrate (TCI) solution as standard prepared at different concentrations (0.1, 0.2, 0.3, 0.4, 0.5, 1 mM) to quantify the TA in the samples. A similar processing and derivatization procedure was applied to the standard solution as the samples.

## Supplementary Information

Supplementary Material 1. Data S1: Figure S1: Genome survey of *T. indica.* Figure S2: Whole genome dot plot. A) *T. indica* vs *C. canadensis* B) *T. indica* vs *G. max*. Figure S3: Schematic diagram of triterpene biosynthesis in plants. Terpene backbone biosynthesis. Figure S4: Alignment of amino acid sequence of the SDH genes of *T. indica*. Supplementary Tables- Table S1: Raw genomic data and transcriptome sequencing data generated in this study for *T. indica*. Table S2: Genome assembly statistics of *T. indica*. Table S3: BUSCO statistics of genome assembly of *T. indica*. Table S4: Summary statistics of repetitive regions of *T. indica* genome identified using RepeatMasker. Table S5: Functional annotation of *T. indica* protein-coding gene set. Table S6: COG categories assigned to coding genes of *T. indica*. Table S7: Identified OSCs in the genome of *T. indica*. Table S8: KEGG pathways assigned to the genes AED categories of *T. indica*. Table 9: KEGG pathways assigned to the genes Sub categories of *T. indica*. Table S10: Species used for phylogenetic analysis and genome annotation. Table S11: Calibration points considered for the divergence time phylogeny of *T. indica*, related to Figure 2A. Table S12: Uniprot IDs of the OSCs from different plant species. Table S13: MZmine 3.9.0 Preprocessing parameters. Table S14: Primer sequence used for RT-qPCR.

Supplementary Material 2. Data S2: Supplementary Table 1: Annotation of highly expanded gene families (≥10 genes). Supplementary Table 2: Expression profile, classification and Ka/Ks values of segmentally duplicated gene pairs. Supplementary Table 3: Metabolite list of flower buds. Supplementary Table 4: Metabolite list of fruit pulp. Supplementary Table 5: Metabolite list of Leaf. Supplementary Table 6: Metabolite list of Seed.

## Data Availability

Data associated with this study have been deposited at the NCBI SRA database under the BioProject accession PRJNA1217361, BioSample accession SAMN46485370. Moreover, all other data is available from the corresponding author upon reasonable request.

## Abbreviations

AED: Asymmetrically expressed duplicates
BUSCO: Benchmarking universal single-copy ortholog
GC-MS: Gas chromatography mass spectrometry
ITS: Internal transcribed spacer
LAI: The long terminal repeat (LTR)- assembly index
L-IDH: L-idonate dehydrogenase
LPWG: Legume phylogeny working group
LTR: Long terminal repeats
matK: Maturase K
NoDiff: No-difference pairs
OSC: Oxidosqualene cyclase
SDH: Sorbitol dehydrogenase
Sub: Sub/Neo-functionalized pairs
TA: Tartaric acid
WGD: Whole genome duplication
