# Correction: Chromosome‑level genome assembly of *Tamarindus indica* provides new insights into the evolution of triterpenes and tartaric acid biosynthetic pathway

**DOI:** 10.1186/s43897-026-00256-5

**Published:** 2026-08-24

**Authors:** Mitali Singh, Manohar S. Bisht, Abhijith M.G., Shruti Mahajan, Vineet K. Sharma

**Affiliations:** MetaBioSys Group, Department of Biological Sciences, Indian Institute of Science Education and Research Bhopal, Bhopal, India

**Correction: Mol Horticulture 6, 36 (2026)**

**https://doi.org/10.1186/s43897-025-00222-7**

Following the publication of original article (Singh et al. 2026), it is reported that Fig. 3 has a text “chromosome 8” overlapped on the scale bar; in Fig. 5, the tissue images on the GC–MS profile are missing, and in figure caption, panel “C” has been incorrectly referred to as panel “D”.

Figure 3 has been corrected from:

**Fig. 3:**
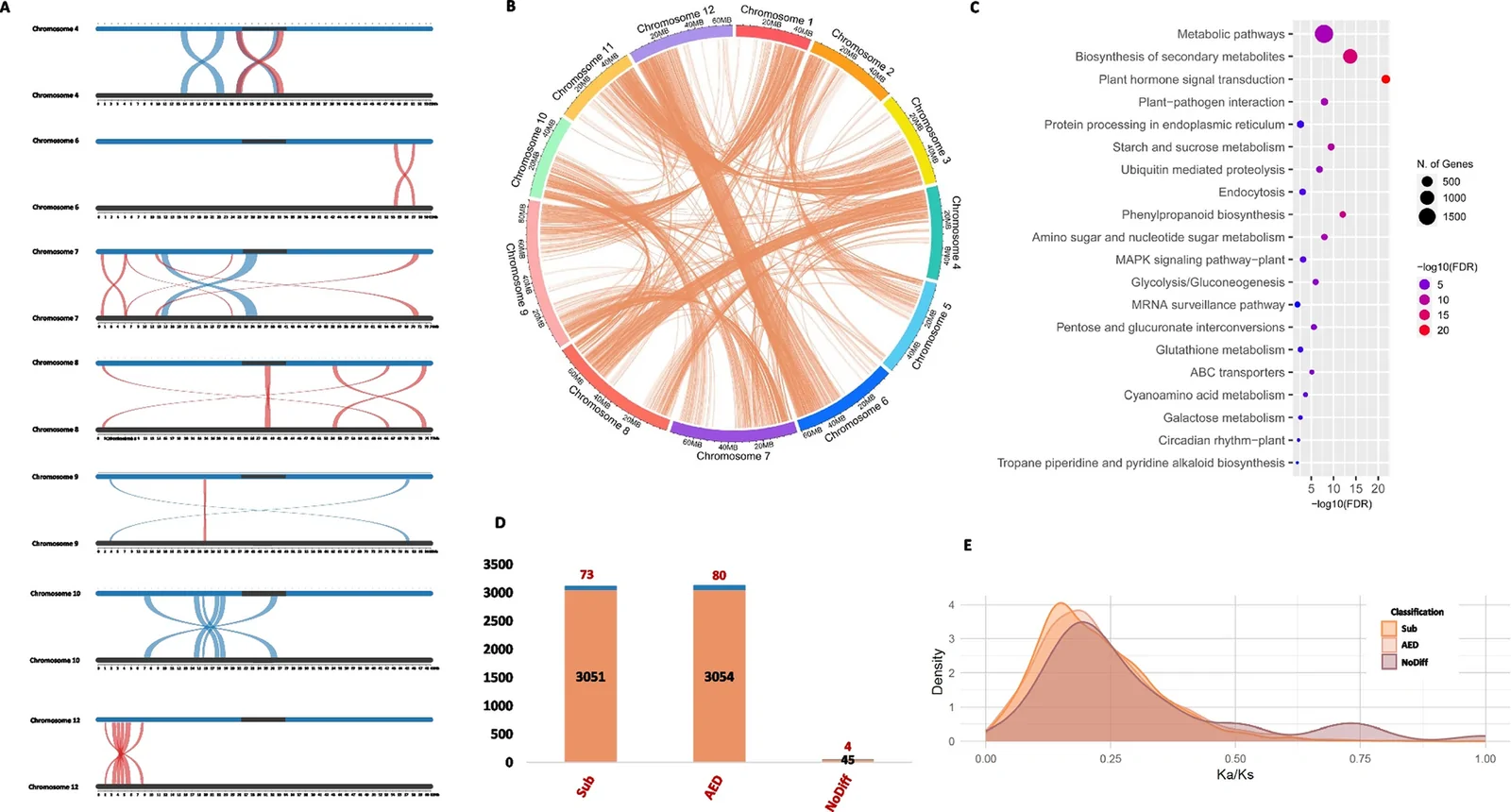
WGD/Segmental duplication and expression analysis. **A** Distribution of intrachromosomal segmental duplication. Blue and red represent forward and reverse matches, respectively, **B** Distribution of interchromosomal segmental duplication, **C** KEGG enrichment of segmental duplicates (adjusted *P-*value < 0.05), FDR: False discovery rate **D** Stacked columns chart showing the ratio of intra to interchromosomal segmental duplicates distribution in AED, Sub, and NoDifff segmental duplicated gene pairs categories, **E** Distribution of Ka/Ks ratio of AED, Sub, and NoDifff segmental duplicated gene pairs. Note: Gene pairs of the chromosome regions are considered

To:

**Fig. 3:**
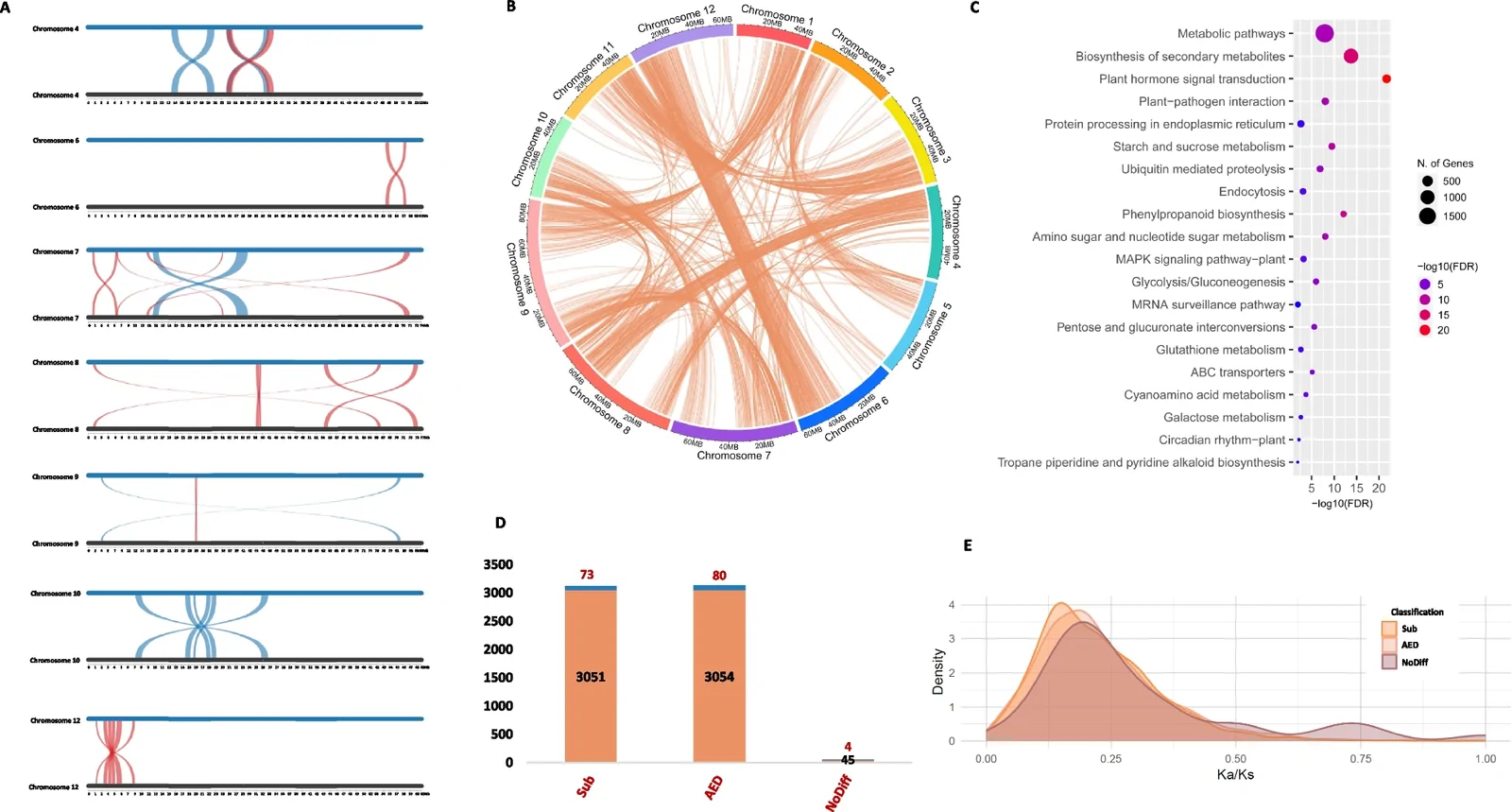
WGD/Segmental duplication and expression analysis. **A** Distribution of intrachromosomal segmental duplication. Blue and red represent forward and reverse matches, respectively, **B** Distribution of interchromosomal segmental duplication, **C** KEGG enrichment of segmental duplicates (adjusted *P-*value < 0.05), FDR: False discovery rate **D** Stacked columns chart showing the ratio of intra to interchromosomal segmental duplicates distribution in AED, Sub, and NoDifff segmental duplicated gene pairs categories, **E** Distribution of Ka/Ks ratio of AED, Sub, and NoDifff segmental duplicated gene pairs. Note: Gene pairs of the chromosome regions are considered

Figure 5 has been corrected from:

**Fig. 5:**
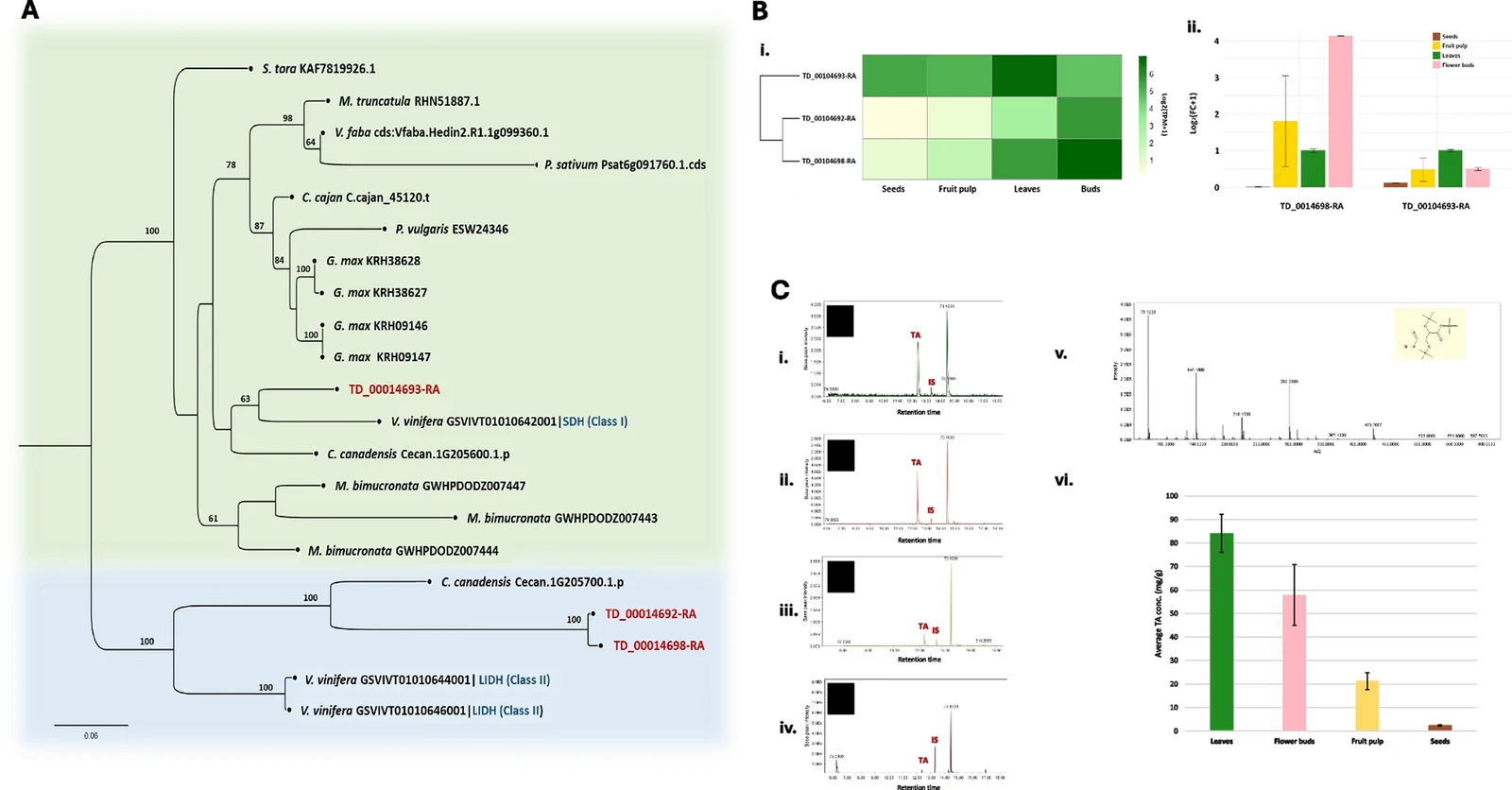
Phylogenetic and gene expression analysis of *SDH* Genes and metabolite profiling of tartaric acid in *T. indica*. **A** The maximum likelihood phylogenetic tree of the *SDH* homolog genes identified in the Legumes species and *Vitis vinifera* (with the *SDH* class marked in blue). *SDH* homologs of *T. indica* are marked in red. Bootstrap values (above 50) are displayed on the nodes, **B** (i) Heat map of the expression value in Log_2_(TPM +1) of the three *SDH* homologs of *T. indica* in the four tissues (*n* = 2) (ii) RT-qPCR analyses of identified *SDH* homologs (TD_00140698-RA and TD_00104693-RA) across four tissues (*n* = 3 biological replicates). Expression levels were normalised using *Elongation factor1-alpha (EF1-α)* as the endogenous control. Gene expression is shown as fold change relative to leaves (reference tissue). Error bars represent the standard error mean (± SEM), **D** GC–MS analysis of the extracts of different tissues of tamarind: (i-iv) Total Ion Chromatogram (TIC) showing tartaric acid-TMS derivative (TA) peak at retention time 12.3–12.5 min and internal standard (IS) peak at 13.3–13.4 min. Peak intensity is mentioned on the y-axis. v. Mass spectra of TA-TMS derivative. vi. The bar plot representation of the TA concentration in each tissue in mg/gram dry weight of the tissue

To:

**Fig. 5:**
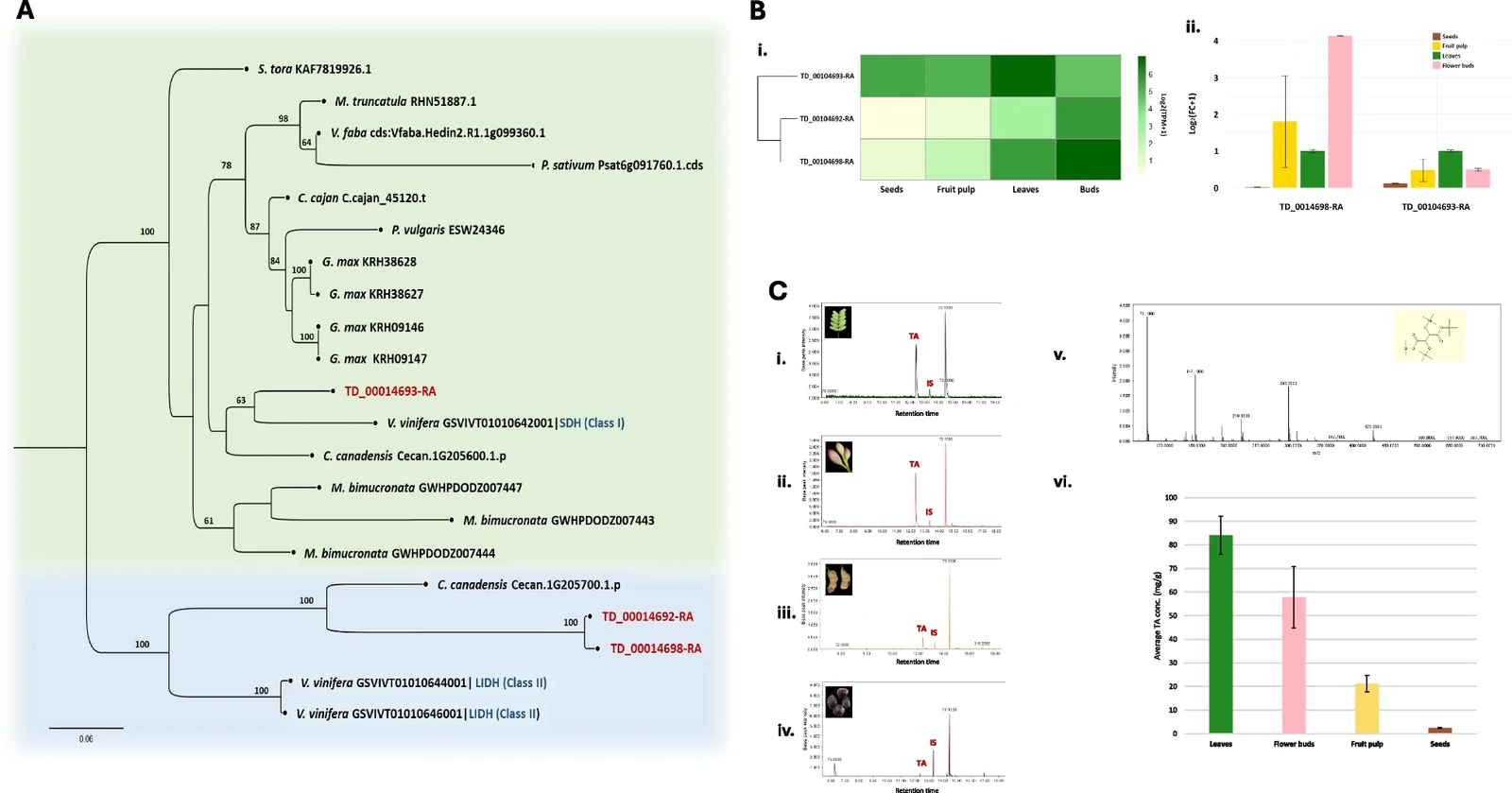
Phylogenetic and gene expression analysis of *SDH* Genes and metabolite profiling of tartaric acid in *T. indica*. **A** The maximum likelihood phylogenetic tree of the *SDH* homolog genes identified in the Legumes species and *Vitis vinifera* (with the *SDH* class marked in blue). *SDH* homologs of *T. indica* are marked in red. Bootstrap values (above 50) are displayed on the nodes, **B** (i) Heat map of the expression value in Log_2_(TPM +1) of the three *SDH* homologs of *T. indica* in the four tissues (*n* = 2) (ii) RT-qPCR analyses of identified *SDH* homologs (TD_00140698-RA and TD_00104693-RA) across four tissues (*n* = 3 biological replicates). Expression levels were normalised using *Elongation factor1-alpha (EF1-α)* as the endogenous control. Gene expression is shown as fold change relative to leaves (reference tissue). Error bars represent the standard error mean (± SEM), **C** GC–MS analysis of the extracts of different tissues of tamarind: (i-iv) Total Ion Chromatogram (TIC) showing tartaric acid-TMS derivative (TA) peak at retention time 12.3–12.5 min and internal standard (IS) peak at 13.3–13.4 min. Peak intensity is mentioned on the y-axis. v. Mass spectra of TA-TMS derivative. vi. The bar plot representation of the TA concentration in each tissue in mg/gram dry weight of the tissue

The original article (Singh et al. 2026) has been corrected.
