# Supplementary material for: Chromosome-level genome assembly of Tamarindus indica provides new insights into the evolution of triterpenes and tartaric acid biosynthetic pathway
Source: Mol Hortic. 2026 May 13;6:36. doi: 10.1186/s43897-025-00222-7 (PMC13169614; doi:10.1186/s43897-025-00222-7)
Supplement: Supplementary file 1 — Supplementary Material 1. Data S1: Figure S1: Genome survey of T. indica. Figure S2: Whole genome dot plot. A) T. indica vs C. canadensis B) T. indica vs G. max. Figure S3: Schematic diagram of triterpene biosynthesis in plants. Terpene backbone biosynthesis. Figure S4: Alignment of amino acid sequence of the SDH genes of T. indica. Supplementary Tables- Table S1: Raw genomic data and transcriptome sequencing data generated in this study for T. indica. Table S2: Genome assembly statistics of T. indica. Table S3: BUSCO statistics of genome assembly of T. indica. Table S4: Summary statistics of repetitive regions of T. indica genome identified using RepeatMasker. Table S5: Functional annotation of T. indica protein-coding gene set. Table S6: COG categories assigned to coding genes of T. indica. Table S7: Identified OSCs in the genome of T. indica. Table S8: KEGG pathways assigned to the genes AED categories of T. indica. Table 9: KEGG pathways assigned to the genes Sub categories of T. indica. Table S10: Species used for phylogenetic analysis and genome annotation. Table S11: Calibration points considered for the divergence time phylogeny of T. indica, related to Figure 2A. Table S12: Uniprot IDs of the OSCs from different plant species. Table S13: MZmine 3.9.0 Preprocessing parameters. Table S14: Primer sequence used for RT-qPCR. [file 43897_2025_222_MOESM1_ESM.docx]

**Title:** **Chromosome-level genome assembly of *Tamarindus indica* provides new insights into the evolution of triterpenes and tartaric acid biosynthetic pathway**

**Authors:** Mitali Singh^1+^, Manohar S. Bisht^1+^, Abhijith M G^1^, Shruti Mahajan^1^, Vineet K. Sharma^1*^

**^+^:** Equal contribution

**Affiliation:**

^1^MetaBioSys Group, Department of Biological Sciences, Indian Institute of Science Education and Research Bhopal, Bhopal, India

*Corresponding Author email: Vineet K. Sharma - [vineetks@iiserb.ac.in](mailto:vineetks@iiserb.ac.in)

**E-mail addresses of authors:** Mitali Singh - [mitali21@iiseb.ac.in](mailto:mitali21@iiseb.ac.in), Manohar S. Bisht - [manohar21@iiserb.ac.in](mailto:manohar21@iiserb.ac.in), Abhijith M G – [abhijith20@iiserb.ac.in](mailto:abhijith20@iiserb.ac.in), Shruti Mahajan – [shrutib2mahajan@rediffmail.com](mailto:shrutib2mahajan@rediffmail.com)

**SUPPLEMENTARY FIGURES**


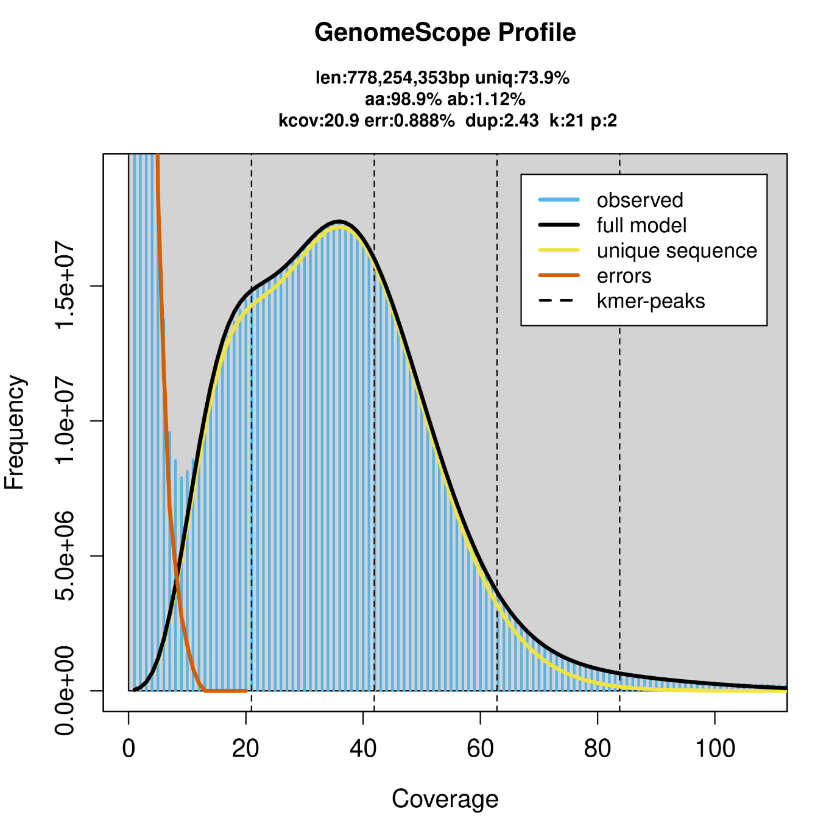


**Figure S1: Genome survey of *T. indica***


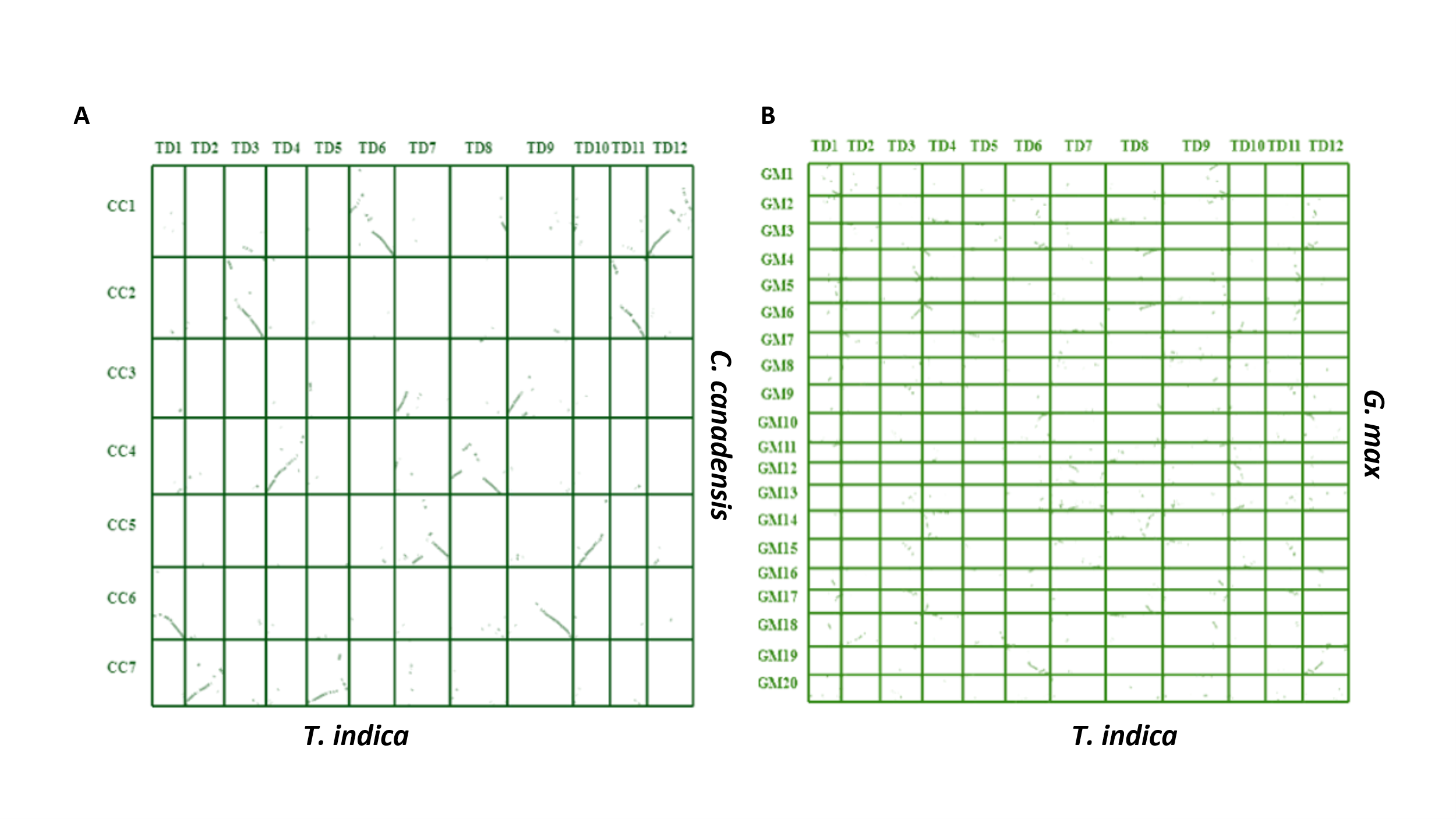


**Figure S2: Whole genome dot plot. A) *T. indica* vs *C. canadensis* B) *T. indica* vs *G. max***

***
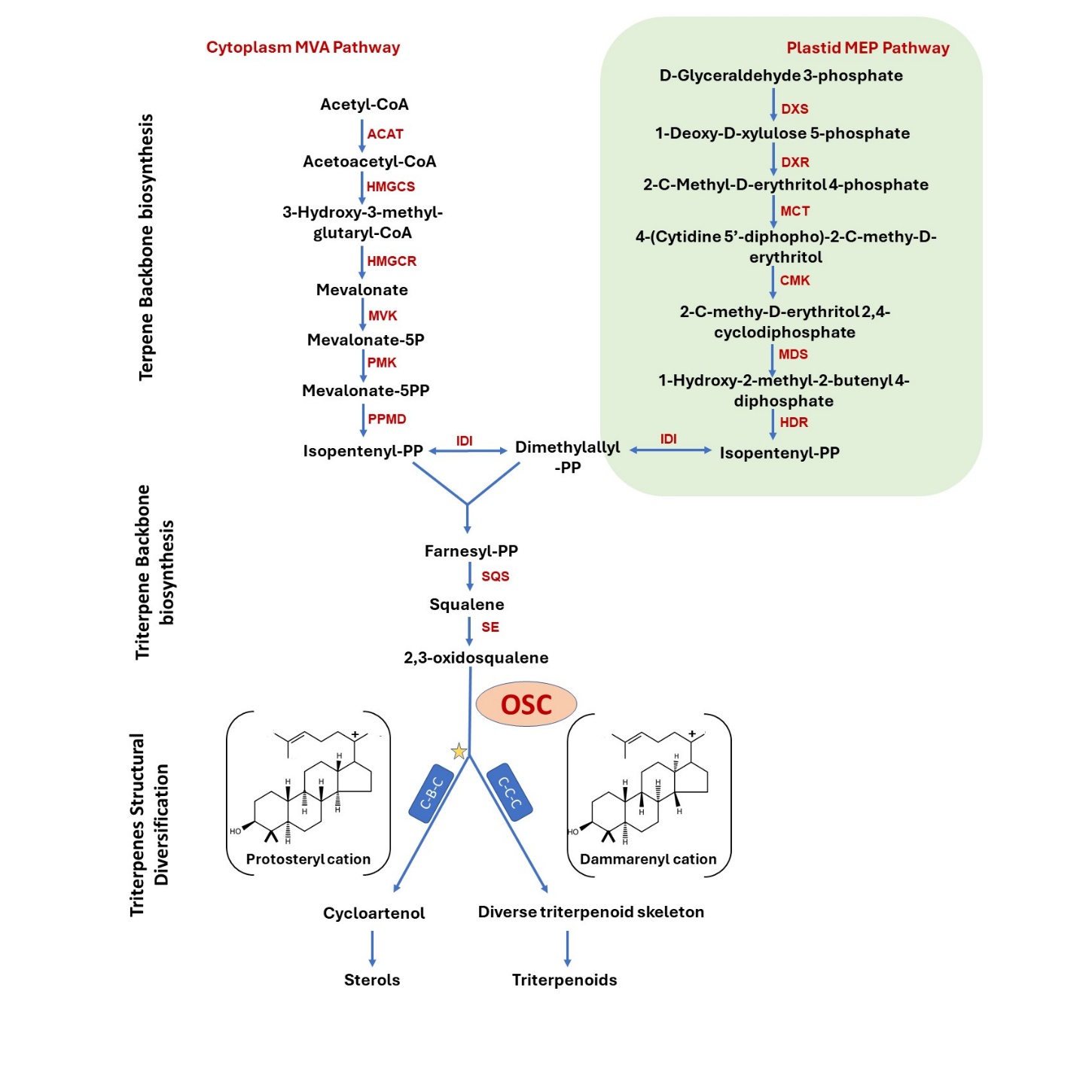
***

**Figure S3: Schematic diagram of triterpene biosynthesis in plants.**

Terpene backbone biosynthesis: MVA pathway: acetyl-CoA C-acetyltransferase (ACAT), hydroxymethylglutaryl-CoA synthase (HMGCS), hydroxymethylglutaryl-CoA reductase (HMGCR), mevalonate kinase (MVK), phosphomevalonate kinase (PMK), and mevalonate diphosphate decarboxylase (PPMD), isopentenyl-diphosphate isomerase (IDI); MEP pathway: 1-deoxy-D-xylulose-5-phosphate synthase (DXS), 1-deoxy-D-xylulose-5-phosphate reductoisomerase (DXR), 2-C-methyl-D-erythritol 4-phosphate cytidyl transferase (MCT), 4-(cytidine-5′-diphospho)-2-C methyl-D-erythritol kinase (CMK), 2-C-methyl-D-erythritol 2,4-cyclodiphosphate synthase (MDS), (E)-4-hydroxy-3-methylbut-2-enyl diphosphate reductase (HDR); Triterpene backbone biosynthesis: squalene synthase (SQS), squalene epoxidase (SE); OSC: 2,3-oxidosqualene cyclase. Star represents the branch point of sterols and triterpenes biosynthesis mediated by OSC.


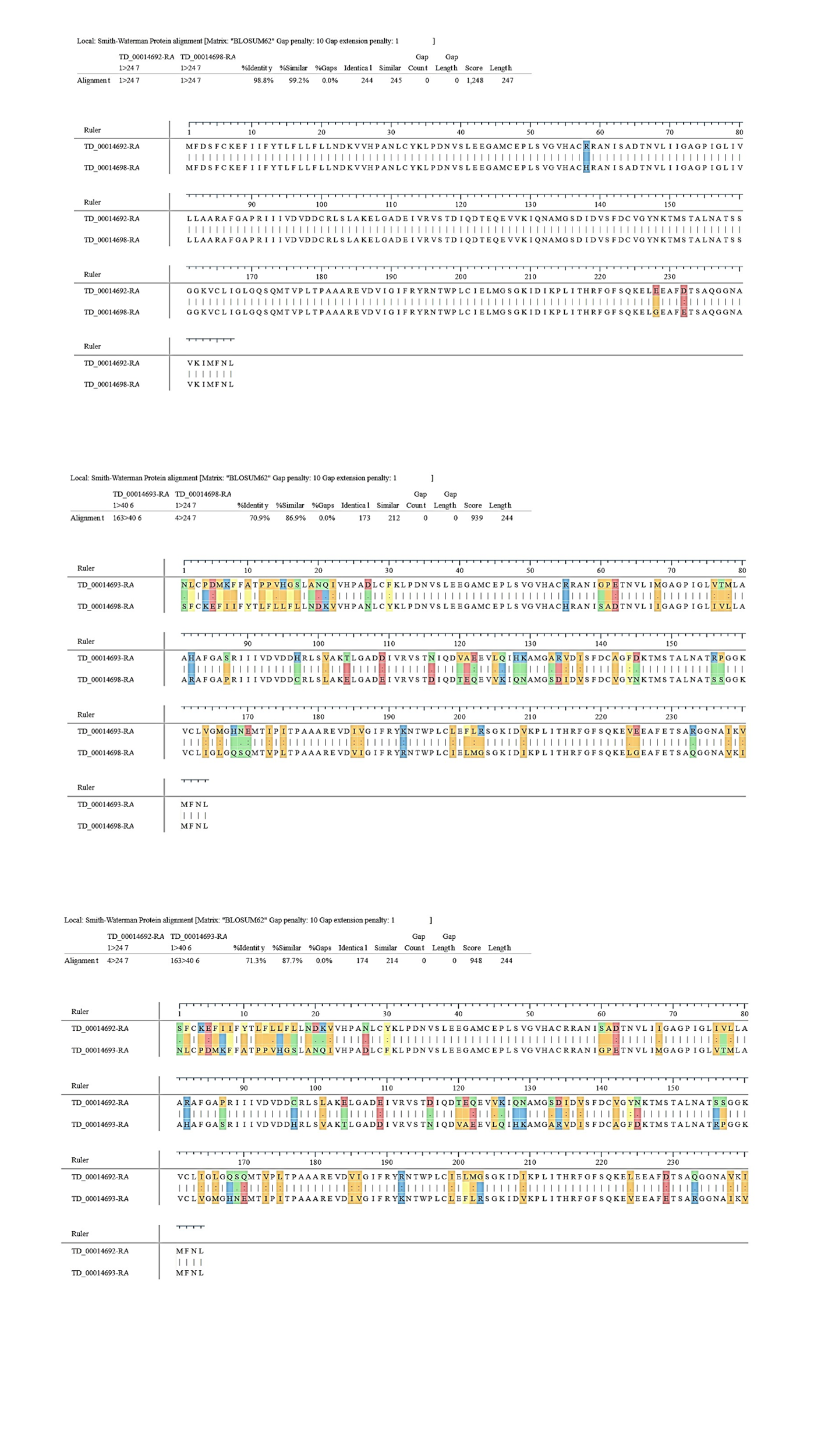


**Figure S4: Alignment of amino acid sequence of the SDH genes of *T. indica***

**SUPPLEMENTARY TABLES**

**Table S1: Raw genomic data and transcriptome sequencing data generated in this study for *T. indica***

| **Raw genomic and transcriptomic sequencing data** | | | |  |
| --- | --- | --- | --- | --- |
|  |  |  |  |  |
| Sequencing data | Total data (bp) | Number of reads | Coverage* |  |
| Oxford Nanopore | 22,417,499,655 | 6,308,410 | 28.81 |  |
| 10X | 60,698,405,502 | 381,750,978 | 78.01 |  |
| HiC | 65,820,733,520 | 412,861,772 | 84.6 |  |
| **RNA-Seq** | | | |  |
| Leaf | 50,33,00,10,640 | 32,34,05,568 | - |  |
| Fruit pulp | 34,89,50,82,740 | 22,18,60,680 | - |  |
| Flower buds | 32,79,83,93,700 | 21,86,55,958 | - |  |
| Seed | 48,52,17,94,200 | 32,34,78,628 | - |  |

*Calculated using the estimated genome size

**Table S2: Genome assembly statistics of *T. indica***

| **Genome assembly statistics** | | | |  |
| --- | --- | --- | --- | --- |
|  |  |  |  |  |
| **Parameters** | 10X assembly | Oxford Nanopore assembly | Final assembly |  |
| Contigs**(>= 0 bp) | 102,645 | 6,301 | 637 |  |
| Contigs (>= 1 Kbp) | 21,650 | 6,177 | 636 |  |
| Contigs (>= 5 Kbp) | 9,967 | 4,461 | 458 |  |
| Contigs (>= 10 Kbp) | 5,127 | 2,808 | 395 |  |
| Contigs (>= 25 Kbp) | 2,812 | 1,286 | 140 |  |
| Contigs (>= 50 Kbp) | 1,975 | 819 | 86 |  |
| Total length (bp) (>= 0 bp) | 883,354,176 | 861,921,070 | 776,716,133 |  |
| Total length (bp) (>= 1 Kbp) | 868,823,962 | 861,834,126 | 776,715,518 |  |
| Total length (bp) (>= 5 Kbp) | 839,294,336 | 856,378,333 | 776,299,047 |  |
| Total length (bp) (>= 10 Kbp) | 805,930,319 | 844,339,796 | 775,855,526 |  |
| Total length (bp) (>= 25 Kbp) | 771,140,477 | 820,822,299 | 772,019,661 |  |
| Total length (bp) (>= 50 Kbp) | 741,866,432 | 804,923,597 | 770,210,279 |  |
| Contigs | 102,645 | 6,301 | 637 |  |
| Largest contig (bp) | 5,761,464 | 19,896,034 | 88,078,137 |  |
| Total length (bp) | 883,354,176 | 861,921,070 | 776,716,133 |  |
| GC (%) | 29.63 | 29.69 | 29.52 |  |
| N50 (bp) | 555,525 | 2,445,853 | 56,663,916 |  |
| N90 (bp) | 13,026 | 183,768 | 43,913,879 |  |
| auN | 866,336.6 | 3,299,738.6 | 58,550,995.3 |  |
| L50 | 395 | 98 | 6 |  |
| L90 | 4,166 | 520 | 12 |  |
| N's per 100 kbp | 1,806.70 | 10.44 | 158.31 |  |

*After length-based filtering by removing scaffold with <5kbp of size. **Contig represents a scaffold

**Table S3: BUSCO statistics of genome assembly of *T. indica***

| **Type of BUSCOs** | **Genome assembly** |
| --- | --- |
| Complete | 92.80% |
| Single (S) | 86.90% |
| Duplicate (D) | 5.90% |
| Fragmented (F) | 0.90% |
| Missing (M) | 6.30% |
| Total BUSCO (n) | 1614 |

**Table S4: Summary statistics of repetitive regions of *T. indica* genome identified using RepeatMasker**

| Total length | 776,716,133 bp | | | | |
| --- | --- | --- | --- | --- | --- |
| GC% | 29.52% | | | | |
| Bases masked | 495,895,020 bp | | | | |
|  |  |  | Number of elements | Length occupied (bp) | Percentage of sequence (%) |
| Retroelements |  |  | 308,370 | 297,004,694 | 38.24 |
|  | SINEs |  | 1,955 | 618,201 | 0.08 |
|  | Penelope |  | 0 | 0 | 0.00 |
|  | LINEs |  | 39,430 | 14,749,439 | 1.90 |
|  |  | CRE/SLACS | 0 | 0 | 0.00 |
|  |  | L2/CR1/Rex | 0 | 0 | 0.00 |
|  |  | R1/LOA/Jockey | 3,318 | 902,816 | 0.12 |
|  |  | R2/R4/NeSL | 0 | 0 | 0.00 |
|  |  | RTE/Bov-B | 5,302 | 2,325,731 | 0.30 |
|  |  | L1/CIN4 | 8,945 | 4,306,250 | 0.55 |
|  | LTR elements |  | 266,985 | 281,637,054 | 36.26 |
|  |  | BEL/Pao | 655 | 267,355 | 0.03 |
|  |  | Ty1/Copia | 101,048 | 81,636,519 | 10.51 |
|  |  | Gypsy/DIRS1 | 156,317 | 195,880,980 | 25.22 |
|  |  | Retroviral | 7,065 | 1,880,072 | 0.24 |
| DNA transposons |  |  | 44,143 | 22,219,256 | 2.86 |
|  | hobo-Activator |  | 67,619 | 20,323,149 | 2.62 |
|  | Tc1-IS630 |  | 38,874 | 8,349,598 | 1.07 |
|  | En-Spm |  | 0 | 0 | 0.00 |
|  | MuDR-IS905 |  | 0 | 0 | 0.00 |
|  | PiggyBac |  | 0 | 0 | 0.00 |
|  | Tourist/Harbinger |  | 2,256 | 1,462,193 | 0.19 |
|  | Other (Mirage, Pelement, Transib) |  | 4841 | 1,181,680 | 0.15 |
| Rolling-circles |  |  | 20101 | 6,997,449 | 0.90 |
| Unclassified |  |  | 317604 | 134,971,895 | 17.38 |
| Total interspersed repeats |  |  |  | 454,195,845 | 58.48 |
| Small RNA |  |  | 963 | 202,178 | 0.03 |
| Satellites |  |  | 0 | 0 | 0.00 |
| Simple repeats |  |  | 187423 | 7,975,423 | 1.03 |
| Low complexity |  |  | 31933 | 1,576,230 | 0.20 |

**Table S5: Functional annotation of *T. indica* protein-coding gene set**

| **Database** | **Annotated** | **Percentage (%)** |
| --- | --- | --- |
| NCBI-nr | 45,710 | 93.5 |
| Pfam | 33,657 | 68.9 |
| Swiss-Prot | 28,327 | 61.97 |
| Total | 45,789 | 93.7 |

**Table S6: COG categories assigned to coding genes of *T. indica***

| **INFORMATION STORAGE AND PROCESSING** | |
| --- | --- |
| **COG category** | **Number of genes** |
| Replication, recombination and repair | 11,140 |
| Transcription | 2,311 |
| Translation, ribosomal structure and biogenesis | 1,125 |
| RNA processing and modification | 923 |
| Chromatin structure and dynamics | 268 |
| **CELLULAR PROCESSES AND SIGNALING** | |
| **COG category** | **Number of genes** |
| Posttranslational modification, protein turnover, chaperones | 2,622 |
| Signal transduction mechanisms | 2,828 |
| Intracellular trafficking, secretion, and vesicular transport | 1,024 |
| Cell cycle control, cell division, chromosome partitioning | 455 |
| Cell wall/membrane/envelope biogenesis | 411 |
| Cytoskeleton | 378 |
| Defense mechanisms | 248 |
| Nuclear structure | 56 |
| Extracellular structures | 63 |
| Cell motility | 4 |
| **METABOLISM** | |
| **COG category** | **Number of genes** |
| Carbohydrate transport and metabolism | 2,010 |
| Lipid transport and metabolism | 1,700 |
| Secondary metabolites biosynthesis, transport and catabolism | 1,220 |
| Inorganic ion transport and metabolism | 1,138 |
| Amino acid transport and metabolism | 1,137 |
| Energy production and conversion | 906 |
| Coenzyme transport and metabolism | 829 |
| Nucleotide transport and metabolism | 252 |
| **POORLY CHARACTERIZED** | |
| COG category | Number of genes |
| Function unknown | 8,724 |

**Table S7: Identified OSCs in the genome of *T. indica***

| **Notation** | **Gene ID** | **Chromosomal location** | **aa residues** | **Assigned funcional class** | **Molecular weight (Da)** | **pI** | **GRAVY** | **Aliphatic index** | **Unstable index** |
| --- | --- | --- | --- | --- | --- | --- | --- | --- | --- |
| TIOSC1 | TD_00015296-RA | 8 | 369 aa | CAS | 42248.74 | 6.53 | -0.173 | 89.84 | 48.38 |
| TIOSC2 | TD_00059514-RA | 10 | 928 aa | CAS | 105810.55 | 7.11 | -0.216 | 85.46 | 45.18 |
| TIOSC3 | TD_00059502-RA | 10 | 828 aa | LUS | 95011.68 | 6.03 | -0.25 | 82.6 | 46.13 |
| TIOSC4 | TD_00051400-RA | 2 | 805 aa | LUS | 93077.69 | 6.38 | -0.299 | 82.78 | 48.04 |
| TIOSC5 | TD_00011171-RA | 8 | 706 aa | LUS | 80409.21 | 6.17 | -0.23 | 85.57 | 48.68 |
| TIOSC6 | TD_00017551-RA | 7 | 795 aa | BAS | 91271.41 | 6.52 | -0.333 | 77.19 | 45.33 |
| TIOSC7 | TD_00046902-RA | 4 | 767 aa | MTT | 88540.61 | 7.14 | -0.286 | 83.95 | 37.98 |
| TIOSC8 | TD_00047602-RA | 4 | 759 aa | MTT | 87608.16 | 6.28 | -0.281 | 82.41 | 36.34 |
| TIOSC9 | TD_00046994-RA | 4 | 512aa | MTT | 58989.77 | 5.86 | -0.157 | 85.57 | 34.58 |

**Table S8: KEGG pathways assigned to the genes AED categories of *T. indica*(Pathways with ≥10 genes are mentioned below)**

| **KEGG Pathways** | **Number of genes** |
| --- | --- |
| Ribosome | 57 |
| Plant hormone signal transduction | 31 |
| Spliceosome | 28 |
| Endocytosis | 25 |
| Protein processing in endoplasmic reticulum | 21 |
| Oxidative phosphorylation | 21 |
| Cysteine and methionine metabolism | 17 |
| Plant-pathogen interaction | 17 |
| mRNA surveillance pathway | 17 |
| Ubiquitin mediated proteolysis | 17 |
| Thermogenesis | 16 |
| Proteasome | 16 |
| Amino sugar and nucleotide sugar metabolism | 15 |
| Glycolysis / Gluconeogenesis | 15 |
| Cell cycle | 15 |
| Nucleocytoplasmic transport | 14 |
| MAPK signaling pathway - plant | 13 |
| Starch and sucrose metabolism | 12 |
| AMPK signaling pathway | 12 |
| Glycerophospholipid metabolism | 12 |
| Lysosome | 11 |
| Carbon fixation by Calvin cycle | 11 |
| Porphyrin metabolism | 11 |
| Glycine, serine and threonine metabolism | 11 |
| RNA degradation | 11 |
| Pyruvate metabolism | 10 |

**Table 9: KEGG pathways assigned to the genes Sub categories of *T. indica*(Pathways with ≥10 genes are mentioned below)**

| **KEGG Pathways** | **Number of genes** |
| --- | --- |
| Plant hormone signal transduction | 38 |
| Protein processing in endoplasmic reticulum | 23 |
| MAPK signaling pathway - plant | 22 |
| Glycolysis / Gluconeogenesis | 18 |
| Endocytosis | 17 |
| Amino sugar and nucleotide sugar metabolism | 16 |
| Starch and sucrose metabolism | 16 |
| Oxidative phosphorylation | 15 |
| Ribosome | 15 |
| Spliceosome | 14 |
| mRNA surveillance pathway | 14 |
| RNA degradation | 14 |
| Circadian rhythm - plant | 13 |
| Pyruvate metabolism | 13 |
| Purine metabolism | 13 |
| Ubiquitin-mediated proteolysis | 13 |
| Carbon fixation by Calvin cycle | 12 |
| Cysteine and methionine metabolism | 12 |
| Plant-pathogen interaction | 11 |
| Glycerophospholipid metabolism | 11 |
| AMPK signaling pathway | 11 |
| Cell cycle | 11 |
| Cysteine and methionine metabolism | 11 |
| Phenylpropanoid biosynthesis | 11 |
| Nucleocytoplasmic transport | 11 |
| Cellular senescence | 11 |
| Phagosome | 11 |
| Glyoxylate and dicarboxylate metabolism | 11 |
| Thermogenesis | 11 |
| Cellular senescence | 11 |
| Galactose metabolism | 10 |
| Glycerolipid metabolism | 10 |
| Proteasome | 10 |
| Lysosome | 10 |

**Table S10: Species used for phylogenetic analysis and genome annotation***

| **Species** | **Accession ID** | **Database** |
| --- | --- | --- |
| *Mimosa bimucronata* | GWHDODZ00000000 | National Genomics Data Center |
| *Senna tora* | GCA_014851425.1 | National Genomics Data Center |
| *Quillaja saponaria* | GCA_029379385.1 | National Genomics Data Center |
| *Acacia crassicarpa* | GCA_034222035.1 | National Genomics Data Center |
| *Cercis canadensis* | ID:705 | Phytozome (V3.1) |
| *Zea mays* | EnsemblPlants (release 59), https://plants.ensembl.org/index.html | |
| *Arabidopsis thaliana* |  |  |
| *Papaver somniferum* |  |  |
| *Nicotiana attenuata* |  |  |
| *Populus trichocarpa* |  |  |
| *Vitis vinifera* |  |  |
| *Manihot esculenta* |  |  |
| *Brassica oleracea* |  |  |
| *Vigna radiata* |  |  |
| *Cajanus cajan* |  |  |
| *Glycine max* |  |  |
| *Medicago truncatula* |  |  |
| *Phaseolus vulgaris* |  |  |
| *Pisum sativum* |  |  |
| *Lupinus angustifolius* |  |  |
| *Trifolium pratense* |  |  |
| *Vicia faba* |  |  |

**Table S11: Calibration points considered for the divergence time phylogeny of *T. indica*, related to Figure 2A**

| **Species pair** | **Class intervals (mya)*** |
| --- | --- |
| *Papaver* - eudicot | 126-132.4 |
| *Nicotiana -Vitis* | 111.4-123.9 |
| *Medicago - Manihot* | 99.0-111.3 |
| *Pisum - Vigna* | 50.3-61.5 |

***** Estimates are taken from: <https://timetree.org/>

**Table S12: Uniprot IDs of the OSCs from different plant species**

| **Species name and Gene name** | **Uniprot ID** |
| --- | --- |
| *Arabidopsis thaliana CAMS1* | P0C8Y0 |
| *Avena strigosa bAS1* | Q93WU1 |
| *Avena strigosa cs1* | Q941S0 |
| *Betula platyphylla CASBPX1* | Q8W3Z4 |
| *Betula platyphylla CASBPX2* | Q8W3Z3 |
| *Betula platyphylla OSCBPW* | Q8W3Z2 |
| *Betula platyphylla OSCBPY* | Q8W3Z1 |
| *Bruguiera gymnorhiza BAS* | A8CDT2 |
| *Bruguiera gymnorhiza LUS* | A8CDT3 |
| *Centella asiatica OSCCCS* | Q6QZW7 |
| *Euphorbia tirucalli EtAS* | Q401R6 |
| *Glycyrrhiza glabra GgbAS1* | Q9MB42 |
| *Glycyrrhiza glabra GgCAS1* | Q9SXV6 |
| *Glycyrrhiza glabra LUS1* | Q764T8 |
| *Hellenia speciosa CSOSC1* | Q8VWY4 |
| *Illex asprella IaAs1* | A0A097A293 |
| *Kandelia candel KcMS* | Q05K37 |
| *Lotus japonicus AMY2* | Q84PE1 |
| *Lotus japonicus OSC3* | Q2WGL7 |
| *Luffa aegyptiaca CAS1* | Q9SLP9 |
| *Malus domestica OSC1* | C7SCX0 |
| *Malus domestica OSC3* | C7SCX2 |
| *Medicago truncatula AMY1* | Q84PE3 |
| *Olea europaea OEA* | A5LHW8 |
| *Olea europaea OEW* | Q9SLW3 |
| *Panax ginseng OSCPNX1* | O82139 |
| *Panax ginseng OSCPNY1* | O82140 |
| *Panax ginseng OSCPNY2* | O82146 |
| *Pisum sativum OSCPSM* | Q9LRH7 |
| *Rhizophora stylosa M1* | A8C980 |
| *Rhizophora stylosa M2* | A8C981 |
| *Ricinus communis LUPS* | Q2XPU7 |
| *Taraxacum officinale TRW* | Q9SLW1 |

| Parameters | GC MS Single Quadrupole |
| --- | --- |
| Mass detection | Noise level: 3.0E2 |
| ADAP chromatogram builder | Min consecutive scans: 5  Min intensity for consecutive scans: 3.0E2  Min absolute height: 3.0E3  *m/z* tolerance: 0.1 *m/z* or 0.0 ppm |
| Chromatogram resolving | Algorithm: ADAP Resolver  Dimension: Retention time  S/N threshold: 7  S/N estimator: intensity window SN  Min feature height: 2.0E3  Coefficient/area threshold: 30  Peak duration range(min): 0.00 - 1.00  RT wavelet range: 0.00 - 0.15 |
| Spectral deconvolution | Algorithm: Multivariate Curve Resolution  Deconvolution window width(min): 0.2  Retention time tolerance(min): 0.04  Min Number of peaks: 1 |
| ADAP Aligner (GC) | Min confidence: 0.1  RT tolerance: 0.15 min  m/z tolerance: 0.1 *m/z* or 0.0 ppm  Score threshold: 0.75  Score weight: 0.1  RT similarity: RT difference |
| Gap filling  (Peak finder) | Intensity tolerance 50%  m/z tolerance: 0.1 *m/z* or 0.0 ppm  RT tolerance: 0.25 min |

**Table S13: MZmine 3.9.0 Preprocessing parameters**

**Table S14: Primer sequence used for RT-qPCR**

| EF1-α | Forward Primer | TATGCCCCTGTGCTTGACTG |
| --- | --- | --- |
|  | Reverse primer | TGGGCTTGGTGGGAATCATC |
|  |  |  |
| TD_00014693-RA | Forward Primer | GTGTTGGTGTGCATGCTTGT |
|  | Reverse primer | ACGATGATGATCCGGGAAGC |
|  |  |  |
| TD_00014692-RA/ TD_00014698-RA | Forward Primer | TGATAAGGTGGTGCATCCGG |
|  | Reverse primer | GACAGGCATGTACACCCACA |
